# GHK-Cu as a Bioactive Metallopeptide and Drug-Delivery Cargo: Coordination Chemistry, Formulation Science, Therapeutic Evidence, and a Translational Roadmap

**DOI:** 10.3390/pharmaceutics18091077

**Published:** 2026-08-27

**Authors:** Diana-Maria Mateescu, Dragos-Mihai Gavrilescu, Ruxandra-Ioana Mincioaga, Ana-Maria Pah, Ana-Olivia Toma, Daniela-Vasilica Serban, Cristiana Adina Avram, Maria-Laura Craciun, Bogdan Enache, Camelia-Oana Muresan

**Affiliations:** 1 Department of Infectious Diseases, Clinical Hospital CF Timișoara, 1 Gării Street, 300166 Timișoara, Romania; diana.mateescu@umft.ro; 2 Department of Orthodontics, Dental District, Zăgazului 3, One Floreasca Vista, Sector 1, 014261 Bucharest, Romania; dr.gavrilescu@outlook.com; 3 Department of Dermatology, Colentina Clinical Hospital, 19-21 Șoseaua Ștefan cel Mare, 020125 Bucharest, Romania; 4 Department of Cardiology, “Victor Babeș” University of Medicine and Pharmacy of Timișoara, Eftimie Murgu Square 2, 300041 Timișoara, Romania; laura.craciun@umft.ro (M.-L.C.); bogdan.enache@umft.ro (B.E.); 5 Center for the Morphologic Study of the Skin (MORPHODERM), “Victor Babeș” University of Medicine and Pharmacy of Timișoara, Eftimie Murgu Square 2, 300041 Timișoara, Romania; daniela.serban@umft.ro; 6 Department of Internal Medicine I, “Victor Babeș” University of Medicine and Pharmacy of Timișoara, Eftimie Murgu Square 2, 300041 Timișoara, Romania; avram.adina@umft.ro; 7 Institute of Legal Medicine Timișoara, 1A Cireșului Street, 300610 Timișoara, Romania; muresan.camelia@umft.ro; 8 Ethics and Human Identification Research Center, “Victor Babeș” University of Medicine and Pharmacy of Timișoara, Eftimie Murgu Square 2, 300041 Timișoara, Romania; 9 Discipline of Forensic Medicine, Bioethics, Deontology, and Medical Law, Department of Neuroscience, “Victor Babeș” University of Medicine and Pharmacy of Timișoara, Eftimie Murgu Square 2, 300041 Timișoara, Romania

**Keywords:** GHK-Cu, copper tripeptide, metallopeptide, coordination chemistry, speciation, labile copper, drug delivery, wound healing, microneedles, critical quality attributes

## Abstract

**Background/Objectives**: Glycyl-L-histidyl-L-lysine (GHK) and its copper(II) complex GHK-Cu have been studied for matrix remodeling, inflammation, redox regulation, angiogenesis, and tissue repair, yet the literature frequently treats GHK-Cu as a single active ingredient despite formulation-dependent variation in coordination state, speciation, stability, pharmacokinetics, and toxicity. We critically evaluate GHK-Cu simultaneously as a bioactive metallopeptide and as a drug-delivery cargo, with explicit separation of apo-GHK, canonical GHK-Cu, GHK-derived copper peptides, and non-GHK copper-peptide systems. **Methods**: We conducted a structured systematic evidence-mapping review of PubMed/MEDLINE, Europe PMC, major publisher platforms, ClinicalTrials.gov, backward citation chains, and official European Union, U.S., and ICH regulatory sources from database inception through 12 August 2026. Biological evidence level and chemical/formulation quality were graded independently using an author-defined two-axis framework. Delivery studies were extracted against a fixed matrix comprising formulation, claimed loading, molar copper occupancy, labile copper, species-resolved release, factorial controls, stability/manufacturability, and objective outcome. Quantitative pooling was not performed because active-entity definitions, formulations, doses, models, comparators, and endpoints were not quantitatively commensurable. **Results**: Preclinical data consistently support effects on matrix remodeling, epithelial repair, inflammatory/redox regulation, and angiogenesis, but the clinical evidence remains sparse and does not meet contemporary active-entity quality standards. Historical cosmetic reports are small or incompletely characterized; a 13-participant post-CO2-laser study was negative on objective endpoints, whereas a 2026 18-participant split-face eyebrow study reported positive cosmetic hair outcomes but did not define GHK-Cu speciation or local exposure. The ongoing phase 2 acute-wound study NCT07437586 remains recruiting and has no efficacy results; its registration cannot be used as evidence of clinical translation. Across delivery studies, particle size, polydispersity, encapsulation efficiency, total peptide/copper content, and bulk release are often reported, whereas molar occupancy, labile copper, and release of intact GHK-Cu versus apo-GHK/free copper are usually not resolved. **Conclusions**: Translation is limited less by biological plausibility than by pharmaceutical definition and evidence attribution. We define the “active pharmaceutical entity” operationally as the reproducible chemical state intended to mediate pharmacology at administration, not as an established regulatory designation or a claim that one immutable molecular species persists in biological fluids. We further propose, explicitly as an author-derived development framework rather than a consensus standard, a control strategy based on molar occupancy, route-specific labile copper specifications, orthogonal speciation, mechanism-linked potency, species-resolved release, factorial controls, route-specific safety decision thresholds, ICH-aligned stability, and GMP-scalable manufacture. Until these requirements are met and controlled clinical efficacy is demonstrated, GHK-Cu should be regarded as a promising but unproven therapeutic cargo rather than a clinically validated regenerative drug.

## 1. Introduction

GHK is a naturally occurring tripeptide composed of glycine, histidine, and lysine. Its capacity to coordinate Cu(II) transformed an initially observed plasma growth-modulating activity into a broader research program spanning copper transport, extracellular-matrix remodeling, inflammation, oxidative stress, angiogenesis, and tissue repair [1,2]. The GHK-Cu literature is unusual in both longevity and heterogeneity: early studies used fibroblast cultures, wound chambers, and animal injury models; later studies added transcriptomic signatures, sophisticated biomaterials, and responsive delivery systems [3,4,5,6,7,8,9,10,11,12,13]. This breadth is scientifically attractive but creates a recurrent interpretive problem. “GHK-Cu” is often treated as a single, self-evident active ingredient even when the administered material, metal-to-ligand ratio, counterions, pH, competing chelators, purity, and free copper content are not fully reported.

For pharmaceutics, these omissions are not peripheral. Coordination state can influence charge, conformation, membrane interaction, chemical stability, biological target engagement, and toxicity. Foundational equilibrium, EPR, X-ray, X-ray absorption, and NMR studies demonstrate that Cu(II)-GHK is pH- and ligand-dependent and that its solution structure is not identical to its solid-state form [14,15,16,17]. Chemical-reactivity and permeation studies further show that peptide–copper behavior is sensitive to redox context, pH, and the biological barrier [18,19,20,21,22]. A formulation may therefore alter not only the rate and site of delivery but also the identity of the species delivered. The relevant pharmaceutical question is not simply whether GHK-Cu is bioactive. It is whether a defined formulation can reproducibly create a therapeutically favorable local exposure to a chemically controlled species while limiting free copper, degradation products, aggregation, and off-target distribution.

The commercial visibility of copper peptides has also produced a disconnect between claim density and evidence quality. Several topical studies report improvements in markers of skin remodeling, but many are small, short, incompletely characterized, conference-based, or conducted with multicomponent products [13,23,24,25]. Conversely, a controlled study after carbon dioxide laser resurfacing did not demonstrate a clear objective advantage for a copper-tripeptide regimen [26]. This mixed human record does not invalidate the molecule; it indicates that formulation, indication, timing, endpoint selection, and exposure confirmation are likely decisive.

Recent work has moved the field from simple creams toward nanoscale carriers, microneedles, pH-responsive polymers, self-assembling systems, electrospun dressings, and injectable depots [27,28,29,30,31,32,33,34,35,36]. A contemporaneous review emphasized synthesis routes, biomanufacturing, quality control, and emerging applications [37], and a further recent review examined the skin-permeability limitations of topically applied GHK and the enhancement strategies proposed to overcome them [38]. The present review is complementary but distinct: it centers on active-entity and speciation control, route-specific formulation science, evidence attribution, regulatory positioning, and stage-gated translational decisions. These platforms create an opportunity to revisit GHK-Cu as a genuine drug-delivery cargo, but they also raise new risks: carrier-driven effects may be misattributed to the peptide; release studies may not distinguish intact complex from copper dissociation; and highly engineered systems may be difficult to manufacture, sterilize, or regulate. A critical synthesis must therefore link mechanism, materials science, analytical chemistry, non-clinical pharmacology, and clinical development.

This review provides that synthesis. Its objectives are to: (i) define precisely what is meant here by the operational “active pharmaceutical entity” and identify the coordination variables that determine it; (ii) grade biological evidence separately from chemical/formulation quality; (iii) distinguish evidence generated with apo-GHK, canonical monomeric GHK-Cu, GHK-derived copper peptides, and non-GHK copper-peptide systems; (iv) evaluate delivery systems according to the barrier they solve; (v) identify analytical and safety requirements for topical, wound, implant, and injectable use; and (vi) propose, as an author-derived decision framework rather than a consensus development standard, a stage-gated translational roadmap tailored to the scope of pharmaceutics.

## 2. Method

This review was revised as a structured systematic evidence-mapping review designed to answer two linked questions: (1) what biological and clinical effects can be attributed to a specified GHK-related entity with reasonable confidence, and (2) whether published delivery systems preserve and measure the same chemical entity they claim to administer. The search was updated from database inception through 12 August 2026. PRISMA 2020 reporting principles were applied where compatible with an evidence map that intentionally includes primary experimental studies, trial-registry records, and regulatory/guidance sources. The protocol was not prospectively registered, and no meta-analysis was prespecified.

PubMed/MEDLINE and Europe PMC were searched using the reproducible core syntax: (“GHK-Cu” OR “GHK Cu” OR “copper tripeptide” OR “glycyl-L-histidyl-L-lysine” OR (GHK AND copper)) AND (“coordination” OR “speciation” OR “labile copper” OR formulation OR “drug delivery” OR liposome OR microneedle OR hydrogel OR polymer OR electrospun OR microsphere OR scaffold OR permeation OR wound OR skin OR pharmacokinetics OR stability OR toxicity OR “clinical trial”). The core query was supplemented by targeted searches pairing the entity terms with individual platform or analytical terms when indexing was incomplete. Major publisher platforms were used to retrieve recent formulation papers and primary reports not consistently indexed under GHK-Cu terminology. ClinicalTrials.gov was searched using GHK, GHK-Cu, copper peptide, and copper tripeptide. Backward citation chaining was performed from foundational and recent reviews, and official European Union, U.S., EFSA, NIH, and ICH sources were searched for product classification, copper-exposure context, stability, and sterile-manufacturing requirements. The date of the final search was 12 August 2026.

Eligible sources were English-language primary studies addressing GHK/GHK-Cu coordination chemistry, analytical characterization, formulation or delivery, ex vivo permeation, mammalian or lower-organism pharmacology, and human efficacy or safety; registered interventional trials; and official regulatory or quality guidance relevant to the proposed product classes. Reviews were used for context and citation chaining but did not substitute for primary results when the primary report was available. Marketing-only claims, duplicate reports, non-informative secondary mentions, and studies of unrelated copper materials without a prespecified comparator role were excluded from evidentiary claims. For entity attribution, each study was assigned to one of four source classes: E1, apo-GHK without deliberate copper complexation; E2, canonical GHK-Cu (nominal or analytically demonstrated Cu(II)-GHK complex); E3, a GHK-derived or chemically modified copper peptide, including dimeric or immobilized derivatives; and E4, a non-GHK copper-peptide or copper-delivery system. Evidence was not transferred across E1–E4 classes unless the text explicitly identified the inference as mechanistic or platform-level rather than GHK-Cu-specific.

Evidence was synthesized with two independent author-defined axes. The biological evidence axis (B1–B5) captures study design and translational proximity: B1, controlled human studies with clinically relevant endpoints; B2, non-controlled human pilot, biomarker, or permeation studies; B3, in vivo mammalian efficacy studies; B4, mechanistic cell, ex vivo, or lower-organism studies; and B5, hypothesis-generating omics, commercial, or conference-level evidence. The chemical/formulation quality axis (C0–C3) captures confidence that the administered entity is chemically interpretable: C3, orthogonally characterized complex with measured stoichiometry/occupancy, bounded labile copper, and species-resolved stability or release appropriate to the claim; C2, chemically supported GHK-Cu identity with at least one orthogonal coordination/speciation measure but incomplete labile copper or release resolution; C1, nominal GHK-Cu based mainly on formulation label, feed stoichiometry, total peptide/copper, or bulk release; and C0, the material is apo-GHK, a derivative/non-GHK copper system, or the active entity cannot be assigned. The two axes are deliberately non-substitutable: a B1 human trial can remain C1 if the test material is poorly characterized, whereas a C3 formulation study can remain B4 if it has no human efficacy evidence.

For every major delivery study, the revision extracted formulation architecture, claimed GHK-Cu loading, whether copper occupancy was measured rather than assumed from feed stoichiometry, whether labile/free copper was quantified, whether release distinguished intact complex from apo-peptide and copper, whether a factorial control set was present, the primary objective outcome, and the availability of stability, sterility, and scale-up information (Table 1). An effect was attributed specifically to canonical GHK-Cu only when both the entity class and experimental design reasonably separated the complex from apo-GHK, free copper, carrier/vehicle, and co-active effects. Table 2 therefore reports biological evidence and chemical/formulation quality separately, rather than collapsing them into a single evidence score.

**Figure 1:**
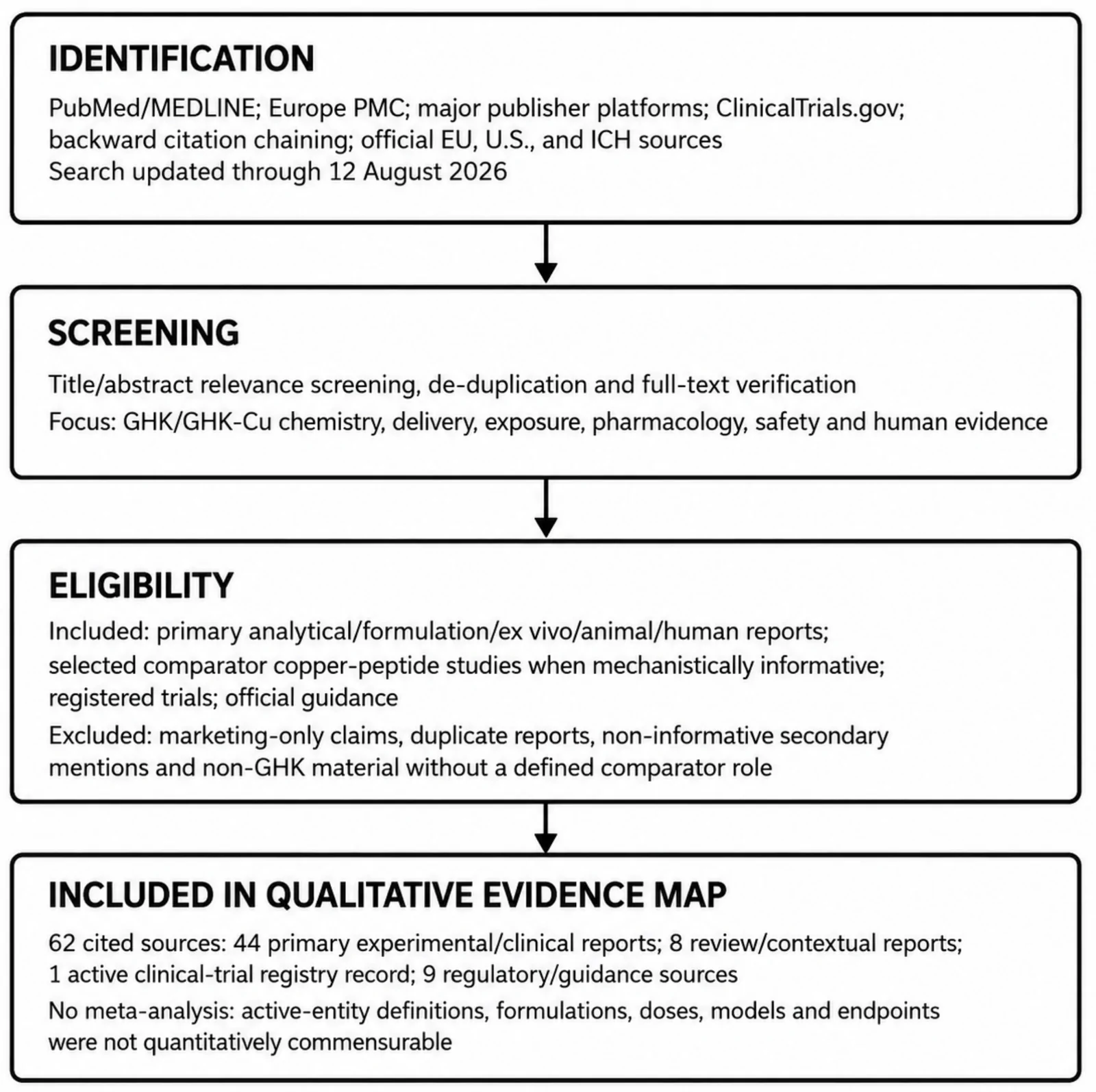
PRISMA-style evidence-selection workflow for the revised systematic evidence map. The figure reports the auditable identification, screening, eligibility, and inclusion logic together with the final 62-source corpus composition. Historical raw database hit counts from the original narrative search were not retrospectively reconstructed; this limitation is explicitly stated in Section 11.

At the revision cutoff, the final qualitative corpus comprised 62 cited sources: 44 primary experimental or clinical reports, eight review/contextual reports, one active clinical-trial registry record, and nine regulatory/guidance sources. The original narrative search had not prospectively retained raw database hit and de-duplication counts. Rather than retrospectively inventing unverifiable PRISMA numbers, the revision documents the auditable selection workflow, exact search logic, eligibility rules, extraction fields, entity classes, dual-axis appraisal, and final source composition in Figure 1 and discloses this residual limitation in Section 11. Quantitative pooling was not attempted because active-entity definitions, dose units, formulations, biological models, comparators, and endpoints were too heterogeneous for a clinically interpretable summary effect. Table 3 summarizes the key pharmaceutical attributes that should be characterized to define the identity, quality, and developability of a GHK-Cu preparation.

## 3. Molecular Identity and Coordination Chemistry

### 3.1. Coordination Motif, Stoichiometry, and Solution Structure

The term “GHK-Cu” compresses a dynamic coordination system into a single label. Classical equilibrium and spectroscopic studies show that GHK binds Cu(II) strongly but that the species present depend on pH, stoichiometry, and competing ligands. Potentiometric and visible-absorption studies identified multiple binary GHK-Cu species across approximately pH 3.5–10.6 and substantial ternary complex formation in the presence of histidine, demonstrating that physiological solution behavior cannot be represented by one immutable structure [14]. At neutral pH, optical/EPR and electron spin-echo measurements support a mononuclear 1:1 Cu(II)-GHK complex with nitrogen coordination that includes the histidyl imidazole; higher-pH transitions alter the coordination environment while retaining imidazole binding [15].

Subsequent multitechnique studies refined this picture. Potentiometry, calorimetry, UV-Vis, circular dichroism, and EPR confirmed multiple stoichiometries and pH-dependent equilibria, with the lysine side-chain amino group contributing mainly at alkaline pH rather than under physiological conditions [16]. X-ray, EPR/HYSCORE, X-ray absorption, and NMR analyses further showed that Cu(II)-GHK is dimeric in the crystal but predominantly monomeric in solution, where the equatorial donor set comprises the N-terminal amino nitrogen, one deprotonated amide nitrogen, and the histidine imidazole nitrogen, while the fourth equatorial position is comparatively labile and can be occupied by an oxygen donor or an exogenous ligand [17]. Fast Cu(II) exchange and detectable higher-order/ternary species reinforce that “1:1 GHK-Cu” is a dominant formulation target, not proof that every copper atom remains in one static coordination microstate [14,15,16,17].

### 3.2. Formulation-Dependent Speciation and Ligand Exchange

For pharmaceutics, the principal consequence is that feed stoichiometry is not equivalent to product speciation. pH controls peptide deprotonation and donor availability; ionic strength changes electrostatic competition; and histidine, albumin, amino acids, chloride, phosphate, citrate, chelators, anionic polymers, proteins, and other excipients can redistribute copper among GHK-bound, ternary, exchanged, or more labile pools [14,16,17]. Reducing agents and antioxidants can alter the Cu(II)/Cu(I) balance, whereas surfactant and lipid interfaces can change local concentrations and ligand accessibility. Dehydration during microneedle casting, electrospinning, lyophilization, or film formation may generate a coordination state that differs from the liquid feed. Accordingly, a nominal 1:1 Cu:GHK preparation should be viewed as a manufacturing input ratio until the finished product is shown to retain the intended occupancy and speciation fingerprint.

The same issue continues after administration. Skin, wound exudate, extracellular matrix, plasma proteins, and intracellular ligands provide competing donors capable of ligand exchange. Such exchange may be pharmacologically relevant rather than simply degradative, but the interpretation changes depending on whether the intended pharmacology is mediated by intact Cu(II)-GHK, a ternary/exchanged complex, apo-GHK after copper transfer, or controlled release of copper. A formulation therefore cannot claim to deliver intact GHK-Cu merely because total peptide and total copper appear in the same receiver compartment; species-resolved measurements are required [21,22].

### 3.3. What We Mean by the “Active Pharmaceutical Entity”

In this review, “active pharmaceutical entity” is an operational development term, not an established regulatory designation for GHK-Cu and not a claim that a single covalent molecular species persists unchanged in biological fluids. It denotes the reproducible chemical state that the product is designed to administer and whose exposure is intended to account for pharmacological activity. For a coordination-dependent metallopeptide, that state must be defined by a set of linked attributes rather than by the name “GHK-Cu” alone: peptide identity and purity; total peptide and total copper; Cu:GHK molar occupancy; a bounded labile/readily displaceable copper fraction; an orthogonal coordination/speciation fingerprint; relevant related species, including apo-GHK and degradation products; and a potency assay connected to the proposed mechanism.

This definition is deliberately falsifiable. If a product loses the intended complex during manufacture or release, its active entity has changed. Conversely, if mechanistic studies demonstrate that therapeutic activity is mediated primarily by controlled copper transfer or by apo-GHK generated after dissociation, the active entity should be redefined to reflect that mechanism rather than preserving “intact GHK-Cu” as a nominal label. The purpose of the definition is therefore not to force one coordination model on all products but to make the chemically administered state explicit enough that batches, formulations, and biological studies can be compared.

### 3.4. Analytical Consequences for Identity and Comparability

A defensible characterization package requires orthogonal techniques because each method interrogates a different part of the equilibrium. UV-Vis and circular dichroism report on the Cu(II) ligand field; EPR/HYSCORE resolves paramagnetic coordination; XANES/EXAFS provides oxidation-state and first-shell structural information; potentiometric titration with speciation modeling establishes protonation-dependent stoichiometry and stability; and ESI-MS or NMR can identify peptide-metal adducts and transformation products [14,15,16,17,18,19]. Element-specific separations such as SEC-, HPLC-, or CE-ICP-MS can distinguish peptide-associated from exchanged copper, while ultrafiltration-ICP-MS, chelator-displacement assays, ion-selective methods, or electrochemical approaches can bound the labile fraction. No single technique should be treated as proof of active-entity identity.

For formulation development, the minimum reporting set should include GHK source and grade, copper salt/counterion, complexation procedure, reaction pH and time, Cu:GHK input ratio, purification or removal of residual unbound copper, final pH and osmolality where relevant, total peptide and copper mass balance, molar occupancy, the method used to quantify labile copper, and at least one orthogonal fingerprint confirming complex formation. The same panel should be applied after manufacturing stresses and during stability/release testing. Without these data, cross-study comparisons of “GHK-Cu dose” remain chemically underdetermined.

## 4. Pharmacodynamic Rationale: What Is Established and What Remains Inferred

Throughout Section 4, Section 5, Section 6 and Section 7, entity provenance is treated as part of the evidence rather than as a footnote. Results obtained with apo-GHK (E1) are not cited as direct evidence for canonical GHK-Cu (E2); GHK-derived copper peptides or immobilized/dimeric constructs (E3) are interpreted as derivative-specific; and unrelated copper-peptide/copper-delivery systems (E4) are used only to illuminate class-level chemistry or carrier behavior. When a mechanistic hypothesis crosses these boundaries, the inference is stated explicitly rather than presented as evidence of interchangeable pharmacology.

**Figure 2:**
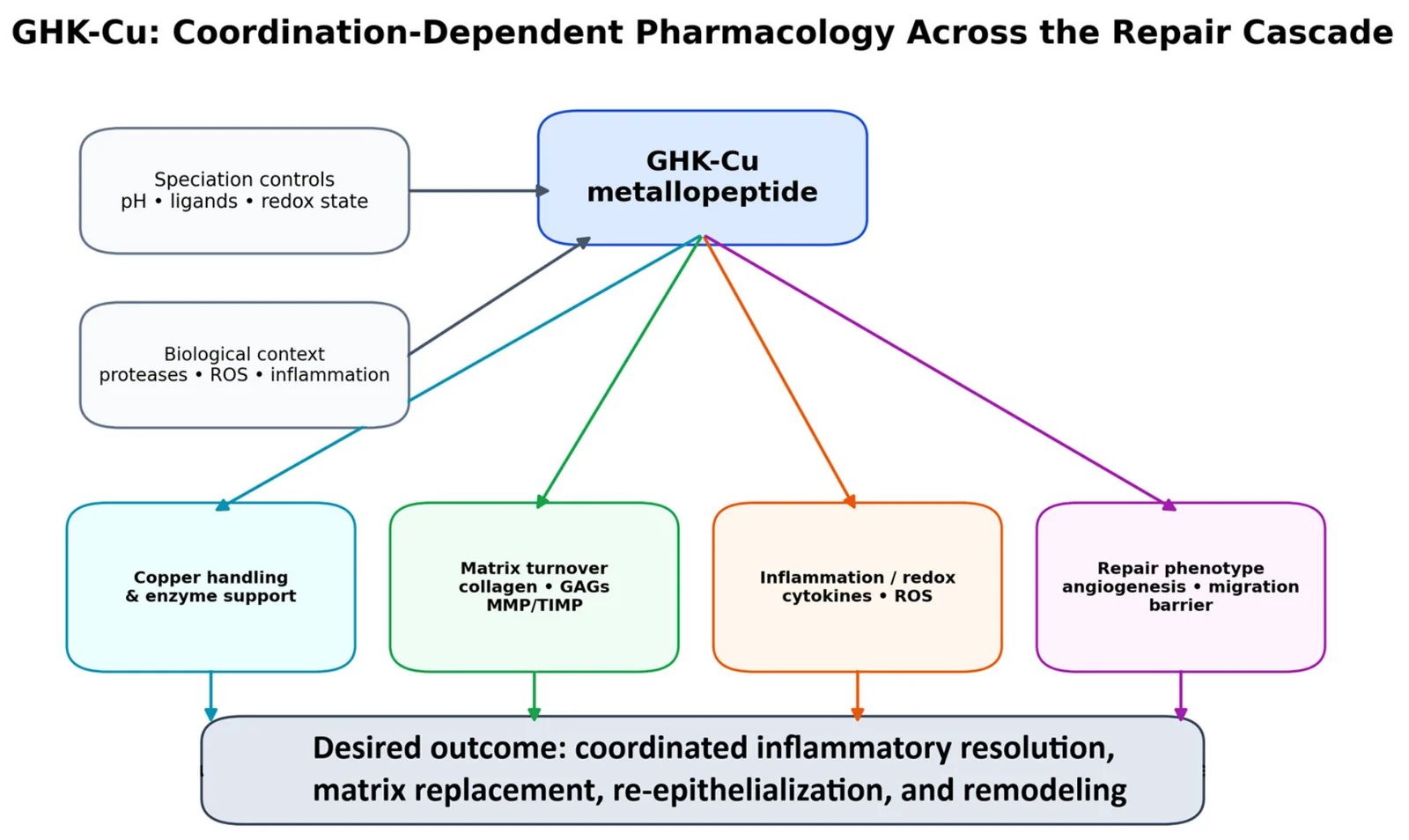
Coordination-dependent pharmacology proposed for GHK-Cu. The diagram separates formulation-controlled inputs from downstream biological programs and highlights the need for temporally coordinated repair rather than maximal stimulation of any single pathway.

The mechanisms attributed to GHK-Cu are numerous, unevenly evidenced, and frequently presented as though they were independent. They are better understood as a set of downstream programs driven by a small number of formulation-controlled inputs, as shown in Figure 2. The subsections below distinguish what the primary literature establishes from what it merely permits; Table 2 then grades the corresponding claims.

**Figure 3:**
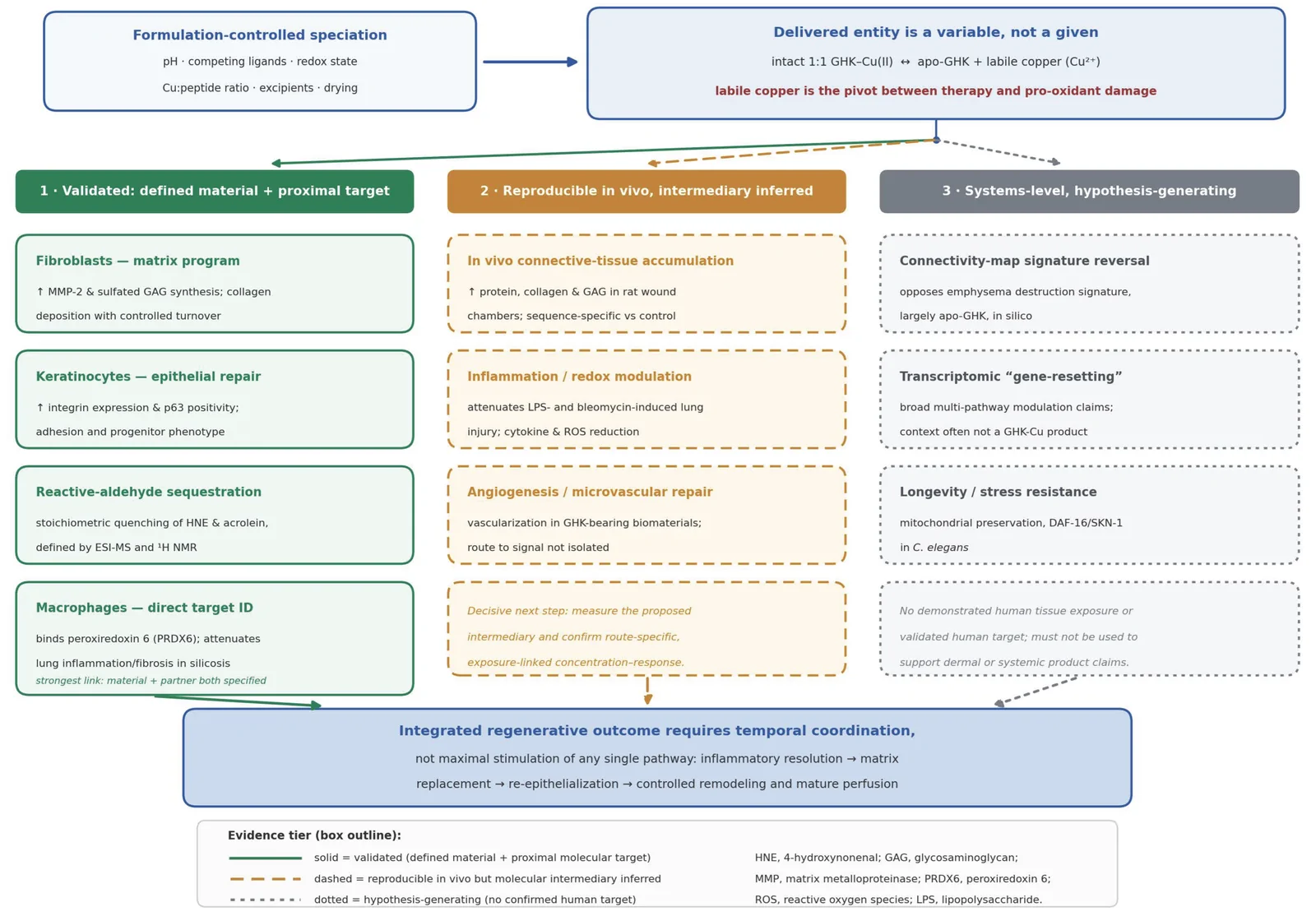
Proposed molecular mechanisms of GHK-Cu stratified by strength of evidence. Formulation-controlled speciation determines the delivered entity, which engages a small set of validated proximal targets (solid outlines: matrix metalloproteinase-2 and glycosaminoglycan synthesis in fibroblasts, integrin and p63 signaling in keratinocytes, reactive-aldehyde sequestration, and peroxiredoxin 6 binding in macrophages), a set of in vivo effects whose intermediaries are inferred (dashed outlines), and systems-level programs that remain hypothesis-generating (dotted outlines: connectivity-map signature reversal, transcriptomic gene-resetting, and DAF-16/SKN-1-associated longevity) [4,5,6,7,9,10,11,12,13,18,19,32,39,41,42,43]. Abbreviations: GAG, glycosaminoglycan; HNE, 4-hydroxynonenal; LPS, lipopolysaccharide; MMP, matrix metalloproteinase; PRDX6, peroxiredoxin 6; ROS, reactive oxygen species.

It is useful to state at the outset which of these mechanisms rest on direct experimental validation and which remain plausible but inferential, because the two are often presented with equal confidence. Three classes can be distinguished. First, effects demonstrated with defined material and a proximal molecular readout: sequence-specific stimulation of matrix metalloproteinase-2 and glycosaminoglycan synthesis in fibroblasts [4,7], induction of integrin and p63 expression in keratinocytes [9], stoichiometric sequestration of the reactive aldehydes 4-hydroxynonenal and acrolein characterized by mass spectrometry and nuclear magnetic resonance [18,19], and—most compellingly—direct target identification of peroxiredoxin 6 by a defined GHK-Cu preparation administered systemically in experimental silicosis [39]. Second, effects that are reproducible in vivo but whose molecular intermediary is inferred rather than measured, such as connective-tissue accumulation in rodent wound chambers [5,6] and attenuation of lipopolysaccharide- or particulate-induced lung injury [11,42]. Third, systems-level claims that are hypothesis-generating only: connectivity-map signature reversal obtained largely with apo-GHK in silico [10], broad gene-resetting narratives [12,13], and lower-organism longevity findings mediated by DAF-16/SKN-1 signaling [43], none of which establishes tissue exposure or a validated human target. Figure 3 maps the proposed molecular pathways onto this hierarchy, and Table 2 grades each claim together with the experiment that would most usefully resolve it. Throughout the subsections below, an effect is described as validated only when both the delivered material and a proximal target were defined.

### 4.1. Extracellular-Matrix Synthesis and Remodeling

GHK-Cu has repeatedly been associated with increased collagen synthesis, glycosaminoglycan production, and remodeling of wounded tissue [3,4,5,6,7]. The most persuasive interpretation is not that the peptide simply “increases collagen” but that it modulates both deposition and turnover. Fibroblast studies reported increased collagen synthesis, while wound models showed changes in matrix metalloproteinases (MMPs), small proteoglycans, and glycosaminoglycans [3,4,5,6,7]. This dual action is biologically plausible because successful repair requires removal of damaged matrix before organized replacement.

Nevertheless, matrix stimulation is context-dependent. In chronic wounds, inadequate deposition coexists with excessive protease activity; in fibrotic disorders, further collagen stimulation may be undesirable. The therapeutic objective should therefore be normalization of repair kinetics and matrix quality, not a universal increase in collagen mass. Formulation studies should measure collagen organization, tensile strength, scar architecture, and the balance between MMPs and tissue inhibitors of metalloproteinases (TIMPs), rather than relying solely on hydroxyproline or a single collagen transcript.

A recent fibroblast and ex vivo skin study reported marked collagen IV upregulation when GHK-Cu was combined with low-molecular-weight hyaluronic acid [23]. The finding is useful as a formulation hypothesis but should not be interpreted as proof of clinical synergy. The ratio-specific response, absence of exposure data, and potential contribution of hyaluronic acid require independent replication with isobolographic or factorial designs.

### 4.2. Keratinocyte Function, Integrins, and Re-Epithelialization

Copper-GHK increased integrin expression and p63 positivity in keratinocyte models [9]. These observations support effects on adhesion, progenitor-like phenotype, and epithelial repair. However, p63 positivity is not a clinical surrogate and prolonged proliferative signaling is not inherently beneficial. The relevant translational endpoints are migration across a wound edge, restoration of stratification, transepidermal water loss, barrier lipid organization, and durability of closure.

For superficial products, epithelial exposure may be adequate even when deep dermal penetration is limited. For chronic wounds, however, the formulation interacts directly with exudate, inflammatory cells, granulation tissue, and microorganisms. The desired release profile may thus differ substantially between intact-skin cosmetic use and open-wound therapy.

### 4.3. Inflammation and Redox Regulation

GHK and GHK-Cu have been linked to sequestration of reactive carbonyl species, modulation of lipid peroxidation, and attenuation of inflammatory signaling [18,19,20]. In a mouse model of lipopolysaccharide-induced acute lung injury, GHK-Cu reduced inflammatory injury [11]. Comparable protection has been reported in bleomycin-induced pulmonary fibrosis [42] and, more informatively, in experimental silicosis, where a defined GHK-Cu preparation attenuated lung inflammation and fibrosis and was shown by chemical-biology target identification to bind peroxiredoxin 6 in alveolar macrophages, without significant systemic toxicity [39]. A 2026 zebrafish-larva study separately reported reduced neutrophil and macrophage migration and lower proinflammatory cytokine expression after copper sulfate or lipopolysaccharide challenge [40]. These findings support anti-inflammatory potential but remain preclinical, use different exposure contexts, and do not establish a human dose window.

Copper is pharmacologically ambivalent: it is an essential cofactor and can support antioxidant enzymes, yet labile copper can catalyze damaging redox reactions. A favorable effect therefore depends on how tightly copper is controlled, where it is released, and the surrounding redox environment. A 2026 analytical study demonstrated laccase-like catalytic activity of GHK-Cu in a defined colorimetric system [44]; this is useful evidence of condition-dependent redox reactivity, not proof of a physiological enzyme-like mechanism. Total antioxidant assays are insufficient because they may obscure pro-oxidant behavior at higher concentrations. Concentration–response studies should include oxidative damage, mitochondrial function, glutathione status, and free copper controls.

The timing of inflammation matters. Early inflammatory signaling supports debridement and host defense, whereas persistent inflammation delays repair. A continuously high local concentration may therefore be less effective than a formulation that provides an early anti-inflammatory but sustained regenerative profile. Responsive hydrogels that release cargo in oxidative or acidic conditions are conceptually well matched to this need, although predictive human wound-fluid models are still lacking.

### 4.4. Angiogenesis and Microvascular Repair

Copper-binding peptides derived from extracellular proteins can stimulate angiogenesis, and GHK-containing biomaterials have been associated with vascularization in repair models [10,32,41]. Angiogenesis is beneficial in ischemic and diabetic wounds but may be undesirable in tumors, proliferative retinopathies, or vascular malformations. Any systemic or injectable program should therefore include context-specific angiogenic safety assessment.

Mechanistically, reported effects may arise from copper-dependent enzymes, matrix remodeling, growth-factor signaling, or macrophage polarization. Few studies separate these routes. A robust development program should combine functional perfusion imaging with endothelial migration, vessel maturity, pericyte coverage, and hypoxia markers, rather than counting CD31-positive structures alone.

### 4.5. Transcriptomic Reversal and Systems-Level Claims

Connectivity-map analyses identified GHK as a compound whose expression signature opposed emphysema-associated lung-destruction signatures [10]. Subsequent reviews proposed broad “gene-resetting” effects [12,13]. A 2026 study in *Caenorhabditis elegans* reported lifespan and stress-resistance effects associated with mitochondrial preservation and DAF-16/SKN-1 signaling [43]. These findings are hypothesis-generating but are frequently overextended: the transcriptomic studies often concern apo-GHK, computational perturbation signatures, or cell contexts that do not match a GHK-Cu formulation, while lower-organism longevity findings do not establish human geroprotective efficacy. None of them establishes tissue exposure or clinical benefit in humans. The instructive exception is the silicosis study, in which a defined preparation was administered systemically and a molecular binding partner was identified directly [39]. The fact that target engagement has been demonstrated only in the setting where both the delivered material and its molecular partner were specified supports the argument of this review rather than contradicting it.

For translational use, omics should be embedded in a causal sequence that runs from a characterized formulation, to measured local exposure, to pathway engagement, and finally to a functional tissue outcome. Prespecified gene modules may be valuable pharmacodynamic biomarkers, but untargeted lists of up- and downregulated genes should not substitute for mechanism. Table 2 summarizes the strength of each major claim discussed in this section together with the experiment that would most usefully resolve it.

### 4.6. GHK-Cu Relative to Other Copper-Binding and Regenerative Peptides

GHK-Cu is one member of a broader class of bioactive peptides, but structural similarity does not establish pharmacological interchangeability. In the nomenclature used here, canonical monomeric GHK-Cu is E2; engineered GHK-derived dimers, conjugates, or surface-immobilized constructs are E3; and copper-binding peptides of different sequence or copper-delivery materials are E4. Table 4 compares these classes to define mechanistic boundaries, not to pool their efficacy. Any biological effect reported for E3 or E4 is therefore treated as derivative- or class-specific unless canonical E2 material was directly tested in the same factorial experiment.

## 5. Biopharmaceutic and Developability Barriers

GHK-Cu is small relative to therapeutic proteins but retains the liabilities of both a peptide and a metal complex. Its small molecular size favors diffusion, yet hydrophilicity and ionization constrain passive partitioning across intact stratum corneum. Proteolysis can reduce intact-peptide exposure in wounds and systemic compartments. Metal exchange can create pharmacologically distinct species. These features make route selection and local delivery central to product design.

The pharmacokinetics of GHK-Cu are best considered as two coupled disposition problems—that of the tripeptide and that of the copper it carries—because the two can separate in vivo. GHK is an endogenous molecule whose plasma concentration is appreciable in early adulthood and declines markedly with age [1,2], and as a small, hydrophilic tripeptide it is a substrate for serum and tissue aminopeptidases and dipeptidyl peptidases; free GHK is therefore expected to have a short systemic half-life, with proteolysis to glycine, histidyl-lysine, and free amino acids as the dominant metabolic route. Copper liberated on dissociation does not behave as a free ion but is rapidly bound by albumin, the exchangeable histidine pool, and ceruloplasmin, enters the systemic copper economy, and is cleared predominantly by hepatobiliary excretion; distribution and elimination of the metal are thus governed by whole-body copper homeostasis rather than by the peptide. Formal absorption, distribution, metabolism, and excretion studies of the intact complex—including radiolabel or stable-isotope mass balance that tracks peptide and copper separately—have not been reported, and this absence is one of the clearest gaps between the biological literature and a translatable product.

Because intact-complex exposure cannot be assumed from administration of a nominal dose, pharmacokinetic characterization must be route-specific and species-resolved. For topical and wound routes the relevant quantities are the local tissue concentration and residence time of the intact complex rather than plasma levels; dermal studies show retention of only a few percent of applied peptide, with permeability coefficients differing by orders of magnitude between isolated stratum corneum and viable epidermis [22], and detection of total copper or total peptide in a receptor compartment does not demonstrate that the intact complex crossed the barrier (Section 8.4). For injectable and implanted depots the dominant pharmacokinetic questions are burst release, depot residence, local persistence, and the systemic copper increment measured against dietary intake and hepatobiliary capacity [35]. The single systemic dataset generated with a defined preparation—the silicosis study, in which GHK-Cu was administered parenterally, engaged peroxiredoxin 6 in alveolar macrophages, and produced no significant systemic toxicity [39]—shows that meaningful tissue exposure of a characterized complex is achievable, but it does not substitute for the biodistribution and repeat-dose toxicokinetics that any systemic or injectable program will require.

Passive skin penetration has been demonstrated in model membranes and in vitro human skin experiments, but retention and flux depend on skin layer, pH, vehicle, and measurement method [21,22]. Detection of total copper or total peptide cannot prove that intact GHK-Cu crossed the barrier. Permeation studies therefore require orthogonal assays and full mass balance; the specific requirements are set out in Section 8.4.

Open wounds remove the stratum-corneum barrier but add exudate dilution, proteases, reactive oxygen species, variable pH, microbial enzymes, and rapid clearance by dressings. A wound product therefore needs sufficient retention and controlled availability rather than maximal permeation. In contrast, an implant coating or injectable depot must minimize burst release and systemic escape while maintaining local activity.

Stability-indicating analysis remains underdeveloped. A validated zwitterionic hydrophilic-interaction chromatographic method has been reported for quantifying copper tripeptide in cosmetics [45], illustrating progress toward product testing. Pharmaceutical development will additionally require separation of apo-GHK, intact complex, related peptides, oxidative products, metal-exchanged species, and aggregates. No single assay is likely to be sufficient.

Finally, dose units must be normalized. Published studies variously report peptide concentration, copper concentration, percentage of a complex, or mass of an undefined ingredient, which makes cross-study dose comparison unreliable even where the underlying experiments are sound. The convention adopted here, and its extension to area-applied products, is specified in Section 8.1.

## 6. Delivery Platforms: Mechanism-Led Selection and Critical Appraisal

**Figure 4:**
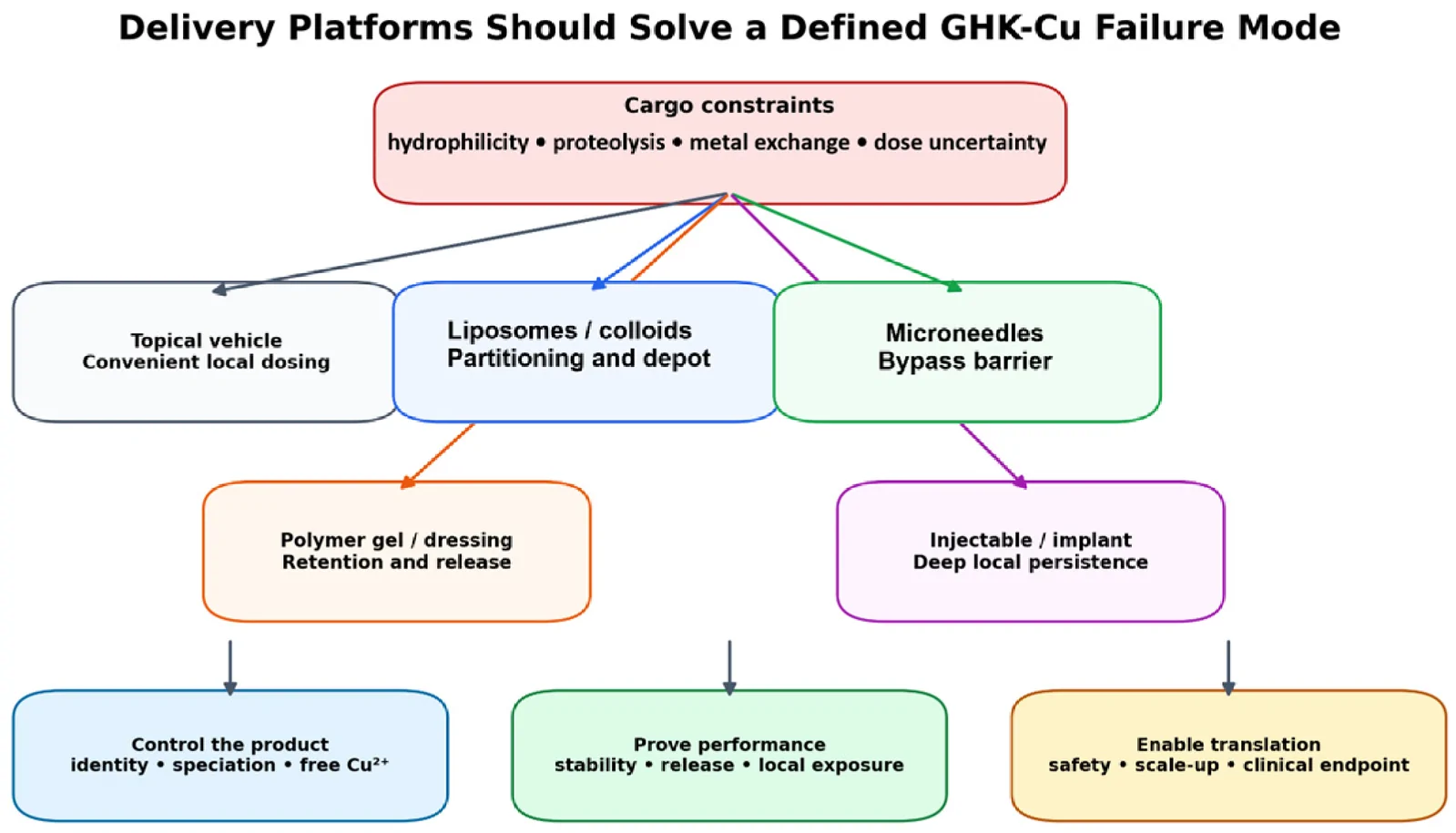
Route and carrier selection should be linked to a defined developability failure mode.

Delivery systems for GHK-Cu are often compared on formulation sophistication rather than on the barrier they are intended to overcome, which makes the literature difficult to navigate. We therefore organize this section by developability failure mode: each platform is introduced in terms of the specific limitation it addresses, as summarized in Figure 4, and is then appraised against the analytical and manufacturing questions it raises. Table 1 provides the study-level systematic extraction requested for cross-platform comparability. Its central finding is that contemporary studies commonly report particle size, PDI, encapsulation or loading efficiency, and bulk release while rarely quantifying molar copper occupancy, labile copper, or species-resolved release. Thus, technically impressive carrier performance frequently remains analytically upstream of Gate 1 (intact-entity release) and Gate 2 (causal attribution) in the translational framework.

### 6.1. Conventional Topical Vehicles

Creams, gels, and serums offer low manufacturing complexity and are appropriate when the target is the superficial epidermis or when repeated application can maintain a local depot. Their weaknesses are variable thermodynamic activity, skin-barrier dependence, susceptibility to complexation by excipients, and uncertain intact-complex delivery. Multicomponent cosmetic products also confound attribution.

An optimized conventional vehicle should be treated as a pharmaceutical formulation rather than a passive base. The design space includes pH, water activity, humectants, penetration modifiers, rheology, preservatives, antioxidant system, and container closure. Excipients should be screened for copper binding, redox effects, and loss of potency. Occlusion may improve hydration and transport but can also increase irritation and alter microbiology.

### 6.2. Liposomes and Other Colloidal Carriers

Liposomes can provide an aqueous compartment for hydrophilic cargo, interact with stratum-corneum lipids, and create a surface depot. Dymek and colleagues produced stable GHK-Cu-loaded liposomes of approximately 100 nm and evaluated lipid composition, bilayer fluidity, encapsulation efficiency, and in vitro tyrosinase and elastase inhibition [27]. The study demonstrates formulation feasibility and offers a strong starting point for a pharmaceutics-oriented program.

However, liposome performance should not be inferred from size alone. Critical attributes include the distribution of GHK-Cu between internal aqueous phase, bilayer interface, and external medium; leakage during storage; lipid oxidation; copper-catalyzed degradation; lamellarity; and changes after application. “Encapsulation efficiency” may be misleading if free external complex remains biologically active or if copper partitions differently from peptide.

Comparative permeation should include free solution at equal thermodynamic activity, blank liposomes, and a disrupted-liposome control. For chronic wounds, liposomes may be embedded in a secondary hydrogel or dressing to improve retention. Microemulsions and ionic-liquid systems may enhance transport, but their surfactant load and irritation potential require careful evaluation [36].

### 6.3. Microneedle-Mediated Delivery

Polymeric microneedle pretreatment increased GHK-Cu permeation through skin models and was reported as effective without evident irritation in the experimental setting [28]. This strategy directly addresses the stratum-corneum barrier and could reduce variability caused by skin condition. It also changes the regulatory and manufacturing problem from a topical product to a drug–device combination.

Two development paths are possible: solid microneedle pretreatment followed by a GHK-Cu formulation, or dissolving/coated microneedles containing the active. The latter offers dose precision but exposes the complex to drying, polymer interactions, and manufacturing stress. Key tests include needle strength, insertion efficiency, dose uniformity, dissolution, intact-complex recovery, microbial control, and pore-closure kinetics. The risk–benefit case is strongest for localized indications where passive delivery is clearly inadequate.

### 6.4. pH-Responsive and Superabsorbent Polymer Systems

Polyaspartic/polyacrylic superabsorbent systems and alginate-containing stimuli-responsive gels have been investigated for controlled GHK-Cu delivery and wound healing [29,30]. These materials combine exudate management with sustained release. Their swelling and ionic groups may also alter copper binding, which can be either useful—by limiting burst release—or problematic—by sequestering the active.

Release testing should therefore quantify intact peptide–copper complex, free copper, and total peptide under sink and wound-relevant conditions. Mathematical fit to a diffusion model is not sufficient if the released species changes over time. Residual monomers, crosslinkers, extractables, mechanical integrity after swelling, and sterilization sensitivity are additional critical attributes.

### 6.5. Self-Assembled, Photo-Crosslinked, and Self-Healing Hydrogels

A hyaluronic-acid hydrogel containing GHK-derived peptide nanofibers was designed for in situ photo-crosslinking and sustained bioactive wound repair [31]. The system improved fibroblast responses, collagen deposition, angiogenesis, and wound closure in preclinical models, with copper complexation enhancing activity. This approach illustrates how self-assembly can increase local retention while the surrounding hydrogel supplies tissue conformity.

A reactive oxygen species (ROS)-responsive hydrogel incorporating a dimeric copper peptide was subsequently reported for diabetic wounds [32]. Dimerization increased protease stability and multivalency, while the hydrogel provided responsive release and ROS scavenging. The system achieved a high degree of wound closure in an infected diabetic-wound model. Importantly, this material is a derivative platform, not necessarily equivalent to monomeric GHK-Cu; its pharmacology and safety must be independently defined.

A food-derived tripeptide–copper self-healing hydrogel has also been developed for infected wound healing [33]. Such systems demonstrate the versatility of copper-coordinated peptides, but they widen the class beyond GHK-Cu. Reviewers and developers should avoid transferring efficacy across peptide sequences merely because copper coordination is shared.

For photo-crosslinked products, photoinitiator identity, light dose, conversion, residual initiator, heat, depth of cure, and peptide photostability are key. For dynamic self-healing gels, rheological recovery, injectability, adhesion, degradation, and batch reproducibility must be tied to release and potency. The hydrogel itself often has antioxidant, adhesive, or immunomodulatory effects; factorial controls are therefore essential.

### 6.6. Electrospun and Composite Dressings

An in situ electrospun poly(vinyl butyral)/poly(vinyl pyrrolidone) smart dressing containing GHK-Cu and an antimicrobial coactive has been reported to combine antioxidant, anti-inflammatory, antimicrobial, and regenerative functions [34]. Multifunctionality is appealing in infected wounds but complicates causal interpretation and regulatory control. The product must demonstrate that each component contributes at a justified dose and that interactions do not destabilize GHK-Cu.

Electrospinning introduces a large surface area and potential rapid release but also solvent, electric-field, and drying stresses. Residual solvent, fiber morphology, dose uniformity, mechanical integrity, moisture response, and release after exudate exposure are required controls. Where an antimicrobial coactive is included, resistance risk and local cytotoxicity should be assessed separately.

### 6.7. Injectable Depots, Fillers, and Implant-Associated Delivery

An injectable hydroxyapatite microsphere filler loaded with GHK-Cu was reported to provide approximately seven days of sustained release, reduce inflammatory and oxidative markers, increase superoxide dismutase activity, and promote collagen deposition in preclinical models [35]. The interest of this work lies in pairing an established biomaterial class with local peptide delivery.

Injectable development nevertheless carries the highest evidentiary burden. Sterility, endotoxin, subvisible particles, aggregation, syringeability, depot migration, local persistence, systemic copper exposure, immunogenicity, and tissue histopathology must be characterized. A carrier intended as a filler may produce mechanical or volume effects that mimic biological improvement. Sham filler, blank microsphere, free GHK-Cu, and free copper controls are necessary.

Implant and scaffold approaches can localize the complex near a regenerative interface. A recent Golgi-targeted copper-delivery strategy for fascia regeneration illustrates how subcellular copper delivery is becoming more precise [46]. Although not a direct GHK-Cu product, it highlights a future direction: carriers may be designed to control not only tissue localization but also intracellular copper trafficking. Such precision should not be assumed for GHK-Cu without direct evidence.

## 7. Human Clinical Evidence and Therapeutic-Domain Translation

The preceding sections argued that the pharmacology of GHK-Cu cannot be separated from its formulation. We therefore begin this section with the complete human clinical record before returning to therapeutic-domain interpretation. This ordering prevents the larger preclinical literature from visually or rhetorically overwhelming the small number of human studies. The subsequent domain subsections identify the highest biological evidence tier, the chemical/formulation quality of the relevant entity, the confounder that most limits interpretation, and the study that would most change a development decision. Table 5 summarizes the domain-level map, whereas Table 5 isolates human evidence.

### 7.1. Human Clinical Evidence: What Has Actually Been Tested in People

Human evidence is sufficiently sparse that it should not be blended with the much larger preclinical literature. The clinical record consists of a small 1998 comparative topical study with ultrastructural endpoints [24], a 2002 conference-level cosmetic report [25], a randomized post-CO2-laser study in which 13 participants completed follow-up and no objective advantage was demonstrated [26], and a 2026 randomized double-blind split-face eyebrow study in 18 healthy participants reporting improved hair count and diameter with a nominal 2% GHK-Cu serum [47]. None of these studies reports the active-entity attributes proposed in Section 3: molar copper occupancy, labile copper, orthogonal speciation, or local intact-complex exposure. Accordingly, their biological evidence level and their pharmaceutical interpretability must be judged separately.

The 2026 eyebrow study is the strongest recent controlled cosmetic signal, but it remains a small single-center study with a cosmetic endpoint and chemically undercharacterized test material [47]. The negative postprocedural study is likewise underpowered but clinically important because it cautions against assuming that robust wound-repair biology will automatically translate into objective human benefit [26]. NCT07437586 [48] is a registered phase 2 randomized vehicle-controlled acute-wound study, but as of the 12 August 2026 search cutoff no efficacy results were posted; trial registration is therefore evidence of ongoing development, not evidence of clinical efficacy or successful translation. Table 5 summarizes the human record separately from the preclinical domain map.

### 7.2. Photoaging and Cosmetic Skin Remodeling

Human efficacy evidence is sparse, heterogeneous, and largely predates the active-entity quality standards proposed in this review. The 1998 human-skin ultrastructural comparison of topical creams [24] included a copper-binding peptide product but did not report molar occupancy, labile copper, intact-complex exposure, or a modern vehicle-controlled dose–response design. Leyden et al. [25] is a conference-level cosmetic report rather than a fully reported controlled trial. Hostynek et al. [22] is an in vitro human-skin penetration study and is therefore treated here as biopharmaceutic evidence, not as a consumer or clinical efficacy study; the 2008 Mazurowska and Mojski report [50] likewise informs penetration/transport rather than therapeutic efficacy. A newly identified 2026 randomized, double-blind, vehicle-controlled split-face study in 18 healthy participants reported that a 2% GHK-Cu serum improved eyebrow hair count and diameter over 12 weeks without serious adverse effects [47]. This study expands the human record but does not validate dermal regeneration or wound-healing claims: it addressed eyebrow hypotrichosis, enrolled only 18 participants, and did not report active-entity speciation, labile copper, local exposure, or mechanism-linked potency. Accordingly, no available human study can yet test whether the quality-control framework proposed here predicts clinical response.

The obstacle in photoaging and cosmetic use is partly structural rather than scientific. Because appearance claims can be made under cosmetic regulation, there is limited commercial incentive to generate medicinal-product-grade efficacy evidence. A developer should therefore choose deliberately between cosmetic claim substantiation, in which instrumental and consumer endpoints may be appropriate, and a medicinal program directed at a defined indication, in which vehicle-controlled dose ranging, a characterized active entity, local-exposure confirmation, and prespecified objective endpoints become mandatory. Presenting historical cosmetic evidence as though it satisfies a therapeutic development standard is precisely the type of category error this review seeks to prevent.

### 7.3. Postprocedural Repair and Acute Wounds

The mechanistic case for acute-wound repair is the best-supported preclinical claim in the literature. Repeated injection into rat wound chambers increased dry weight, total protein, collagen and glycosaminoglycan content in a concentration-dependent manner, with elevated type I and type III collagen messenger RNA and an increased relative proportion of dermatan sulfate, while an inactive control tripeptide produced no effect [5,6]. This sequence-specific, matrix-directed action is reproducible and biologically coherent.

The corresponding controlled human evidence is nevertheless negative on objective endpoints. In a randomized study of skin-care regimens after circumoral carbon dioxide laser resurfacing, computerized analysis and blinded assessors found no significant difference in erythema resolution and no objective advantage in wrinkles or overall skin quality, although patient-reported satisfaction favored the copper-tripeptide arm [26]. Thirteen patients completed the study. A trial of this size cannot exclude a moderate effect, and the products’ active content was not characterized, so the result is best read as a warning against claim inflation rather than as evidence of absence. The endpoint window may also have been mismatched: if the principal action is on matrix organization rather than early inflammation, 12 weeks of erythema scoring may not detect it.

The decisive study is therefore an adequately powered randomized trial following a standardized ablative or fractional procedure, using a defined intact-complex content, a true vehicle control, blinded objective imaging or histology as the primary endpoint, and local-exposure confirmation in at least a subset of participants. Postprocedural skin is a favorable setting because the barrier is transiently compromised, the injury is standardized and scheduled, and follow-up is short. As of 12 August 2026, the phase 2 randomized, double-blind, vehicle-controlled split-wound study of topical GHK-Cu gel in standardized punch-biopsy wounds (NCT07437586) remained recruiting, with no posted efficacy results [48]. The registry entry is therefore evidence that a controlled clinical test is underway, not evidence that GHK-Cu has achieved clinical translation, and it is not used anywhere in this review to support efficacy.

### 7.4. Chronic and Diabetic Wounds

Chronic wounds are the most biologically coherent target, but much of the most sophisticated evidence is not for canonical monomeric GHK-Cu. The pathophysiology—deficient and disorganized matrix deposition, excessive protease activity, oxidative burden, impaired angiogenesis, and persistent inflammation—maps closely onto the actions attributed to GHK-related copper systems, and the requirement for local rather than systemic exposure suits a peptide with poor systemic developability. The delivery literature here spans canonical/nominal GHK-Cu (E2), GHK-derived dimeric copper peptides (E3), and non-GHK copper-peptide systems (E4); these classes are analyzed for platform lessons but are not treated as interchangeable efficacy evidence.

The confounding in this domain is unusually severe because every high-performing system is multifunctional by design. The responsive hydrogel combines a protease-stabilized dimeric derivative with a matrix that itself scavenges reactive oxygen species [32]; the electrospun dressing incorporates a quaternary ammonium antimicrobial alongside the peptide [34]; and superabsorbent systems provide exudate management independently of any peptide effect [29,30]. In each case, reported wound closure is a property of the system, not necessarily of GHK-Cu, and the literature does not currently separate the two. Factorial designs including blank carrier, carrier plus apo-peptide, carrier plus equimolar free copper, and free complex at matched thermodynamic activity are the minimum requirement for attribution and are almost entirely absent.

Human translation faces the additional difficulty that chronic-wound outcomes are dominated by offloading, debridement, revascularization and infection control, which can easily obscure a moderate pharmacological effect. A rational sequence is dose ranging in a standardized infected diabetic model with the full control set, followed by an exploratory human study in which wound-fluid concentrations of intact complex, apo-peptide and labile copper are measured directly, before any attempt at a confirmatory closure trial.

### 7.5. Implant, Scaffold, and Hard-Tissue Applications

A distinct and less crowded opportunity is the use of GHK-related copper chemistry as a surface-conjugated or matrix-embedded regenerative cue rather than as a released drug. However, the studies in this domain span E2–E4 materials: some use GHK-Cu or GHK-functionalized constructs, whereas others use broader copper-peptide or targeted copper-delivery strategies. Their value is therefore mechanistic and platform-specific unless the canonical GHK-Cu entity is directly demonstrated.

The pharmaceutical advantages of this configuration are real: the dose is local and low, the biomaterial supplies retention, and systemic exposure is minimal. The unresolved question is mechanistic and consequential for both regulation and safety. Where the peptide is covalently or electrostatically bound to a surface, it is not established whether the intact complex engages cells or whether the construct functions principally as a slow, chemically buffered copper source. These are different pharmacologies with different toxicological profiles, and distinguishing them requires species-resolved release measurement at the interface rather than bulk copper assay.

### 7.6. Injectable Aesthetic and Soft-Tissue Applications

An injectable filler consisting of GHK-Cu electrostatically adsorbed onto hydroxyapatite microspheres was reported to provide approximately seven days of sustained release with acceptable injectability, reduce inflammatory and oxidative markers, increase superoxide dismutase activity, and promote collagen deposition in preclinical models [35]. This is a notable advance because it links a clinically familiar biomaterial class to local peptide delivery. However, the pharmacological contribution of GHK-Cu cannot be inferred from filler performance alone, and the separate 2026 zebrafish inflammation study [40] should not be treated as validation of this depot formulation.

It is also the domain with the highest evidentiary burden and the least tolerance for the analytical gaps described in Section 5. A filler produces mechanical and volumetric effects that can mimic biological improvement, so sham filler and blank microsphere arms are indispensable. Sterility, endotoxin, subvisible particulate matter, aggregation, syringeability, depot persistence and migration, local histopathology and systemic copper exposure must all be characterized, and adverse events are difficult to reverse once a depot is placed. Where injectable copper-peptide preparations are supplied outside an approved marketing authorization, they sit outside any defined evidentiary or quality framework; the applicable rules on compounded and unapproved peptide preparations differ substantially between jurisdictions and change frequently and should be confirmed against current national guidance rather than inferred from the literature.

### 7.7. Non-Dermal and Systemic Claims

A separate group of claims concerns pulmonary, neurological, skeletal, oncological, and longevity applications, resting largely on connectivity-map analyses, transcriptomic reviews, lower-organism experiments, and rodent models [10,11,12,13,39,42,43]. The tripeptide reversed a gene-expression signature of emphysematous destruction in silico and recapitulated transforming-growth-factor-beta-associated patterns in fibroblasts [10], and the complex attenuated lipopolysaccharide-induced acute lung injury in mice [11]. The pulmonary evidence is the strongest of this group: independent rodent studies report benefit in bleomycin-induced fibrosis [42] and in silicosis, the latter with direct identification of a binding partner and with no significant systemic toxicity [39]. The *C. elegans* study adds mechanistic evidence in a lower organism but does not establish a systemic human indication [43]. These are legitimate research directions, not near-term products: route-appropriate pharmacokinetics, biodistribution, repeat-dose toxicology, and exposure-linked efficacy in humans remain absent, and even the pulmonary work has not been carried into a defined inhaled or systemic product with a specified labile copper limit. A computational signature obtained with apo-GHK in a lung-derived cell context cannot support claims for the cutaneous exposure achieved by a cream.

## 8. Analytical Control, Quality Attributes, and Safety Requirements by Route

Section 3 and Section 5 argued that the identity of the delivered species is a formulation variable. That position has direct analytical consequences, because a control strategy built on assay of peptide and assay of copper cannot detect the changes that matter most. This section sets out the quality attributes, methods, and safety studies that a pharmaceutical program would need, and Table 6 assigns them by route. The intention is not to impose drug-grade requirements on cosmetic products but to make the difference between the two explicit, so that the evidence generated matches the claim made.

### 8.1. Defining and Releasing the Active Entity

A defensible release specification for a GHK-Cu drug product should combine peptide identity and purity, total peptide content, total copper content, the derived molar occupancy, a limit for labile or readily displaceable copper, a characteristic spectroscopic or chromatographic fingerprint, a potency assay linked to the intended mechanism, related substances including apo-peptide and oxidative products, water content, and counterion identity. The visible d–d absorption band of the Cu(II) complex at 580–600 nm provides a convenient orthogonal confirmation of complex formation, is sensitive to ligand exchange, and has been used to characterize monomeric and dimeric copper peptides [32].

Chromatographic control of the intact complex is feasible. A validated zwitterionic hydrophilic-interaction method with ultraviolet detection, coupled to fabric-phase sorptive extraction, has been reported for the quantification of copper tripeptide in cosmetic creams, with satisfactory linearity, limits of detection and quantitation, and separation of the analyte from cream components within ten minutes [45]. Preformulation characterization of the native peptide, including solubility and degradation behavior, is also available [51]. Pharmaceutical development would extend to a stability-indicating configuration capable of resolving apo-GHK, the intact complex, related peptides, oxidative products, metal-exchanged species, and aggregates. No single method is likely to be sufficient, and an orthogonal panel should be assumed.

Dose units require the same discipline. The preferred primary unit is the molar concentration of intact GHK-Cu, supported by total peptide, total copper, and labile copper. For dressings, scaffolds, and other area-applied products, dose per unit area, released dose per unit area and residual dose should be reported, since concentration alone is uninterpretable for such systems.

### 8.2. Speciation and Control of Labile Copper

Labile copper is the single most consequential attribute in this product class, because it determines the boundary between the therapeutic and the pro-oxidant behavior of the same element. Ultrafiltration or size-based separation followed by inductively coupled plasma mass spectrometry, chelator-displacement colorimetric assays, ion-selective measurement, and electron paramagnetic resonance each provide partial information; used together they can bound the free fraction adequately for specification setting. The specification itself should be set on toxicological grounds and will differ by route, being most restrictive for injectable and implanted products and least restrictive for a rinse-off or intact-skin topical product.

Because speciation is formulation-dependent, excipient compatibility screening must include copper-binding and redox endpoints rather than appearance and peptide recovery alone. Phosphate, citrate, aminopolycarboxylate chelators, amino-acid buffers, anionic polymers, and proteins compete with GHK for copper; reducing agents and antioxidants shift the Cu(II)/Cu(I) balance; surfactants and lipid interfaces alter local concentration; and drying steps in film, fiber or microneedle manufacture can generate species not present in the liquid feed. Each of these should be treated as a potential critical process parameter and evaluated with a speciation-sensitive readout.

### 8.3. Stability-Indicating Methods and Degradation Pathways

Four degradation or transformation routes should be assumed and characterized: peptide-backbone hydrolysis under acidic and alkaline conditions; oxidation of the peptide and copper-catalyzed oxidation of excipients; metal exchange with competing ligands; and physical aggregation or particle formation. The demonstrable laccase-like activity of GHK-Cu in a defined analytical system further supports treating catalytic redox behavior as condition dependent [44]. A forced-degradation program covering pH extremes, peroxide challenge, thermal stress, photolytic exposure, and, where relevant, freeze–thaw should achieve mass balance across intact complex, apo-peptide, degradation products, and total copper. A method that reports only loss of the parent peak is insufficient because complex dissociation can occur without peptide loss.

Container-closure evaluation deserves particular attention. Copper adsorption to and leaching from primary packaging, oxygen ingress, headspace volume, and light transmission all influence the labile copper trend over shelf life. Photostability testing should account for both the metal-centered chromophore and the photochemistry of the imidazole side chain.

Beyond forced degradation, the stability-indicating method must be exercised across the storage conditions the product will actually encounter. The three-month accelerated dataset specified at Gate 1 is deliberately an early development screen, not an ICH-complete registration stability package. For a conventional medicinal drug product, ICH Q1A(R2) recommends an accelerated study at 40 °C ± 2 °C/75% RH ± 5% RH for 6 months with at least three time points (for example, 0, 3, and 6 months), together with long-term data sufficient to support the intended shelf life [52]. GHK-Cu programs should use the 3-month result as an early stop/redesign checkpoint, then continue to the full ICH-aligned program before pivotal clinical or registration supply. At every stability time point, peptide assay and total copper should be accompanied by molar occupancy, labile copper, an orthogonal speciation fingerprint, degradation products, potency, pH, and dosage-form-specific physical/microbiological attributes. Photostability should be assessed in accordance with ICH Q1B [53], because both the peptide and the metal-centered coordination environment may be light-sensitive. For liposomes, fibers, hydrogels, and depots, the same program should also follow size/PDI or morphology, rheology/mechanics, container-closure compatibility, leakage/burst release, and species-resolved release so that an apparently stable total copper assay cannot mask loss of the intended active entity.

### 8.4. Biorelevant Release and Confirmation of Exposure

Release testing should reflect the environment at the applied site rather than a convenience medium. For wound products this implies simulated wound fluid containing albumin at physiological concentration, physiological chloride and phosphate, and, for stimulus-responsive systems, a defined oxidative or acidic challenge. Release should be reported in a species-resolved manner—intact complex, apo-peptide, and labile copper as functions of time—because a construct can display textbook diffusion-controlled release of total copper while releasing a chemically different entity at each stage. Fitting a diffusion model to total copper data does not address this.

For dermal products, permeation studies should use orthogonal assays with full mass balance, distinguishing donor depletion, stratum-corneum retention, viable epidermal and dermal deposition, receptor-phase recovery, and degradation products. The available literature is instructive in both directions: complexation increases the migration rate of copper through membranes modeling the intercellular lipid barrier, and the distribution of species is pH-dependent [21,50]; measured permeability coefficients differ by four orders of magnitude between isolated stratum corneum and isolated epidermis, with retention of only a few percent [22]. A recent methodological appraisal concluded that the transport of liposome-encapsulated GHK-Cu in particular remains poorly characterized [54]. Detection of total copper or total peptide in a receptor compartment cannot establish that the intact complex crossed the barrier, and claims of dermal delivery should not be made on that basis. For injectable and implanted products, in vitro release should be complemented by measurement of local tissue and systemic copper.

### 8.5. Non-Clinical Safety by Route

Topical products on intact skin require irritation, sensitization, and phototoxicity assessment, with attention to copper-associated contact sensitization in susceptible individuals and to the possibility that penetration-enhancing vehicles increase immunologically relevant exposure. Wound products additionally require cytocompatibility in wound-relevant cells at clinically achievable concentrations, demonstration that granulation and re-epithelialization are not impaired, and confirmation that host antimicrobial defense is preserved. Injectable and implanted products carry the full parenteral burden: sterility assurance, endotoxin, visible and subvisible particulate matter, aggregation under stress, syringeability/injection force, depot persistence and migration, local histopathology, systemic copper toxicokinetics, and product-level hypersensitivity/immunogenicity assessment.

No validated GHK-Cu-specific human NOAEL, dermal exposure limit, or labile copper safety threshold exists. To make the roadmap operational without presenting unsupported clinical limits, we therefore propose conservative development decision specifications rather than dosing recommendations. For products claiming the nominal 1:1 complex, lot release should target a Cu:GHK molar occupancy of 0.95–1.05. The readily labile/free copper fraction should initially be controlled to <=5 mol% of total copper for topical medicinal and wound products and <=1 mol% for injectable or implanted products, measured with a validated displacement or separation method. These values are author-proposed analytical starting specifications, not established toxicological thresholds; any oxidative injury, sensitization signal, or in vivo copper accumulation at exposures meeting them would require tighter limits. Drift above the provisional limit during stability or biorelevant release testing is a stop/redesign criterion because it indicates that the administered species no longer matches the claimed active entity.

A second threshold concerns systemic copper burden. EFSA concluded that no copper retention is expected at total oral intake of 5 mg/day in adults, while the U.S. adult tolerable upper intake level is 10 mg/day [55,56]. These dietary values are contextual ceilings and cannot be converted directly into parenteral limits. For chronic topical or wound programs, we propose a conservative early-development trigger that worst-case product-derived systemic absorption should remain below 0.5 mg copper/day (10% of the EFSA safe oral level) unless product-specific toxicokinetic data justify otherwise. Injectable and implant programs should not use this oral-derived trigger as a safety limit; they require route-specific repeat-dose toxicology and toxicokinetics that separately track peptide and copper, establish a NOAEL/exposure margin, and demonstrate absence of progressive hepatic, neurological, renal, or depot copper accumulation. Across all routes, efficacy concentrations should be bracketed by a preclinical safety margin that includes oxidative damage, mitochondrial function, glutathione status, and context-specific angiogenic safety, because total antioxidant assays can conceal pro-oxidant behavior at higher concentrations [13,57]. Angiogenic programs should explicitly consider malignancy, proliferative retinopathy, and vascular malformation as contexts requiring heightened caution [41,49].

### 8.6. Long-Term Toxicity, Immunogenicity, and Regulatory Safety Considerations

The safety considerations most relevant to clinical use extend beyond the acute, route-specific studies of Section 8.5 to the consequences of repeated or prolonged exposure. The dominant long-term concern is cumulative copper burden. Chronic or large-area topical use, repeated injection, or long-dwell implants should be evaluated for hepatic and, at high exposures, neurological copper accumulation, interpreted against whole-body copper handling but not justified solely by oral dietary limits [55,56]. Repeat-dose toxicology of appropriate duration—with toxicokinetics that track peptide and copper separately and with histopathology of liver, kidney, nervous system where relevant, and the local depot—is therefore a prerequisite for any systemic, injectable, implanted, or chronically applied medicinal product. Genotoxicity is expected to be low for the tripeptide itself, but copper-catalyzed oxidative endpoints should be included, and reproductive/developmental assessment should follow the intended indication and population.

Immunogenicity and hypersensitivity require explicit attention despite the small size of the peptide. Copper is a recognized contact sensitizer in susceptible individuals, and a penetration-enhancing vehicle or a breached barrier increases immunologically relevant exposure, so delayed-type hypersensitivity and metal-allergy screening are warranted for wound, implant, and injectable products. The peptide is unlikely to be strongly immunogenic on its own, but engineered derivatives, conjugates, and particulate carriers can act as adjuvants or present repetitive epitopes; immunogenicity risk should therefore be assessed at the level of the finished construct rather than the free complex [32,34]. For implanted and depot products, foreign-body response, local granuloma formation, and the durability of any device component belong in the same assessment, which should be proportionate to route, dwell time, and reversibility.

These requirements should be scoped to the regulatory status the product will claim, developed further in Section 9. A cosmetic marketed under intact-skin appearance claims carries a safety-assessment and claims-substantiation burden but not a therapeutic one; a medicinal product, device, or combination requires a non-clinical safety package matched to route and duration, a labile copper limit set on toxicological grounds, and pharmacovigilance appropriate to an irreversible depot where relevant. The practical recommendation is that the long-term toxicity, immunogenicity, and copper-exposure assessment be specified at the same time as the intended claim and route, so that the safety evidence generated matches the use for which authorization will be sought rather than being retrofitted after a formulation is fixed.

### 8.7. Manufacturing Scale-Up and Commercial Translation

A recurring omission in the GHK-Cu literature is whether a promising prototype can be made reproducibly under a pharmaceutical quality system. The pharmaceutically decisive operation is not peptide synthesis alone but complexation and its preservation through downstream processing. Molar ratio, copper salt/counterion, reaction pH and time, order and rate of addition, mixing, temperature, purification of unbound copper, hold times, drying, crosslinking, electrospinning, filling, and container closure should be treated as candidate critical process parameters because they can shift molar occupancy, labile copper, aggregation, and the speciation fingerprint. Scale-up should therefore be managed through a CQA-CPP control strategy with in-process tests that are sensitive to speciation, not merely separate peptide and total copper assays.

Sterility strategy must be selected early for wound, injectable, and implanted products. Terminal heat, irradiation, or other terminal processes are acceptable only if a validated cycle preserves peptide integrity, copper occupancy/speciation, potency, carrier structure, and species-resolved release. If terminal sterilization causes degradation or metal exchange, aseptic manufacture becomes the more defensible route, with a documented contamination-control strategy, qualified cleanroom/process controls, environmental monitoring, process simulation, container-closure integrity, and endotoxin/particulate control. EU GMP Annex 1 explicitly frames sterility assurance as a system of contamination control rather than reliance on a final sterility test alone [58]. Most published GHK-Cu carrier studies do not report a validated sterilization method, poststerilization speciation comparability, or a pharmaceutical aseptic-process strategy; this absence is made explicit in Table 1.

Manufacturability should be weighed alongside biological performance. Conventional vehicles and some polymer gels are comparatively scalable; liposomes require batch control of size distribution, lamellarity, leakage and lipid oxidation; microneedles and electrospun dressings introduce drying, electric-field, mold/fiber uniformity, residual-solvent, and device-performance constraints; photo-crosslinked hydrogels require control of light dose, conversion and residual initiator; and injectable depots require the tightest aseptic/sterility, particulate, syringeability, migration, and labile copper specifications. Gate 1’s three-month accelerated study is therefore a prototype-screening milestone only. Products intended for medicinal development should progress to ICH-aligned long-term/accelerated stability and engineering/validation batches that demonstrate batch-to-batch and post-scale/site-change comparability using the same orthogonal speciation panel used for release [52,53]. A platform that cannot reproduce its active-entity fingerprint, release profile, potency, microbiological quality, and key carrier CQAs at scale should not advance to confirmatory clinical development.

## 9. Regulatory Positioning: Cosmetic, Medicinal Product, Device, or Combination

The same molecule can fall under at least four regulatory categories, and the status determines the evidence burden far more decisively than the underlying science does. Clarity on this point is a prerequisite for rational development, and its absence explains a good deal of the mismatch between claim density and evidence quality described in Section 1.

In the European Union, a finished product containing copper tripeptide may be marketed as a cosmetic only when its intended purpose and claims fall within the cosmetic definition and the product satisfies Regulation (EC) No 1223/2009, including safety assessment, product information, notification, and claims substantiation [59]. The regulation does not constitute therapeutic approval of GHK-Cu as an active substance. In the United States, product status likewise depends substantially on intended use as established by claims, consumer perception, and other evidence; disease-treatment or structure/function claims can cause a product presented as a cosmetic to be regulated as a drug [60].

A GHK-Cu medicinal product would require a quality dossier of the kind outlined in Section 8, route-appropriate non-clinical safety, and clinical evidence for a defined indication. In the European Union, the medicinal-product/device boundary depends on the principal intended action. Under Regulation (EU) 2017/745, a device that incorporates a substance which, if used separately, would be a medicinal product and whose action is ancillary remains subject to device requirements plus consultation on the substance’s quality and safety [61]. Certain injectable soft-tissue fillers without an intended medical purpose fall within Annex XVI, with common specifications established by Commission Implementing Regulation (EU) 2022/2346 [61,62]. The classification of microneedles and drug-loaded dressings depends on their design, claims, and principal mode of action rather than on the presence of GHK-Cu alone.

The practical implication is that the regulatory pathway should be selected before the evidence program is designed. An ancillary-substance device route may be efficient for some dressings but constrains the medicinal claims made for the peptide; a medicinal route permits mechanism-based therapeutic claims but requires a substantially more demanding analytical, non-clinical, and clinical package. Jurisdiction-specific classification and current guidance should be confirmed for the final formulation and claims, particularly for injectable, compounded, or otherwise unapproved peptide preparations.

## 10. An Author-Proposed Stage-Gated Translational Roadmap

The roadmap in this section is an author-proposed translational heuristic constructed from the evidence gaps identified in this review. It is not an ICH, FDA, EMA, ISO, pharmacopeial, or professional-society development standard, and its gates and provisional specifications should not be interpreted as validated regulatory acceptance criteria. Its purpose is narrower: to make explicit the order in which we believe entity definition, formulation feasibility, causal attribution, exposure-linked in vivo efficacy, exploratory human evidence, and confirmatory clinical testing should be addressed for GHK-Cu. The framework is written for local-delivery programs in wound repair or postprocedural skin, which appear most tractable on current evidence, and must be adapted to route, claim, product classification, and jurisdiction. Table 7 summarizes these author-proposed decision points.

Within this author-proposed framework, Gate 0 concerns the entity. Before any formulation work, the program should possess a characterized active with a defined counterion, established molar occupancy, an orthogonally confirmed complex, a validated labile copper method with a provisional route-appropriate limit, a mechanism-linked potency assay, and a standardized reporting set covering peptide source and grade, copper salt, complexation procedure, reaction pH and time, molar ratio, purification, residual unbound components, and final pH. If labile copper cannot be controlled within the provisional limit across the intended formulation pH range, the program should stop or change the chemistry rather than proceed on the assumption that the biology will compensate.

Within this author-proposed framework, Gate 1 concerns formulation feasibility. The minimum deliverables are a prototype with defined loading and, for colloidal systems, distribution of the complex between internal phase, interface, and external medium; species-resolved release in a biorelevant medium; a three-month accelerated stability checkpoint with mass balance and the complete active-entity panel; and excipient compatibility assessed against redox and copper-binding endpoints. The three-month checkpoint is an early development stop/go screen and does not replace the six-month accelerated/long-term program expected for medicinal registration under ICH Q1A(R2) [52]. A prototype that releases predominantly labile copper, loses complex integrity during manufacture, or shows material drift in occupancy/speciation should be redesigned at this stage, which is where drying, electrospinning, crosslinking, and depot-loading operations frequently reveal hidden comparability problems.

Gate 2, as proposed here, concerns attribution and addresses the step most often absent from the published literature. The deliverable is a factorial in vitro and ex vivo dataset comparing the formulated complex against blank carrier, carrier plus apo-peptide, carrier plus equimolar free copper, and free complex at matched thermodynamic activity, with an exposure–response relationship for at least one mechanism-linked endpoint. If the blank carrier reproduces most of the effect, the program is a biomaterials program rather than a peptide program and should be redirected accordingly. This is a reframing rather than a failure, but it must be recognized before clinical investment.

Gate 3, as proposed here, concerns in vivo proof of local exposure and functional effect. The deliverables are an animal model matched to the intended indication, direct measurement of local intact-complex concentration over the dosing interval, and a functional outcome—matrix architecture and mechanical properties, perfusion and vessel maturity, or barrier restoration—rather than a single biomarker or transcript. Programs should stop if efficacy cannot be demonstrated at exposures that a human formulation could plausibly achieve and should include the angiogenic and oxidative safety screens described in Section 8.5.

Gate 4, as proposed here, concerns human exploratory evidence: a small, vehicle-controlled study in a standardized setting, with a biopsy or imaging biomarker and a prespecified pathway module rather than an untargeted gene list, together with confirmation of local exposure and formal tolerability assessment. Only at Gate 5 does a confirmatory trial become rational: adequately powered, indication-specific, with an objective primary endpoint and endpoint timing matched to the mechanism. A program that reaches Gate 5 with the preceding deliverables in hand will be in a substantially stronger position than the current literature, in which claims of this magnitude rest on studies of a dozen participants.

## 11. Limitations of the Evidence Base and of This Review

Several limitations should be considered. The revised manuscript is a structured systematic evidence map rather than a prospectively registered systematic review or meta-analysis. The original work began as a narrative state-of-the-art search, and raw database hit, duplicate-removal, and exclusion counts were not prospectively retained. We therefore did not retrospectively fabricate PRISMA numbers. Instead, the exact search logic, source classes, eligibility criteria, entity taxonomy, study-level extraction fields, dual biological/chemical appraisal axes, and final corpus composition are now explicit, and Figure 1 shows the auditable selection workflow. Study selection and the author-defined B/C assignments still require judgment, so selection and interpretation bias cannot be excluded. Quantitative pooling was not attempted because active-entity definition, formulation, dose, biological model, comparator, and endpoint heterogeneity would make a pooled effect estimate clinically misleading rather than merely imprecise.

The search was restricted to English-language sources and indexed databases supplemented by publisher platforms, reference lists, trial registries, and regulatory documents. Commercial reports, conference abstracts, and industry-sponsored evaluations are unevenly retrievable, and their omission is likely to be non-random; in a field with a large consumer market, this may understate unpublished negative or equivocal work. Publication bias probably favors positive carrier studies because systems that fail to improve on free solution are less likely to be reported. The ongoing phase 2 wound study remained without posted efficacy results at the 12 August 2026 cutoff and therefore cannot be used to support efficacy or clinical translation [48].

A further structural limitation is that many primary studies do not report the parameters argued here to be decisive—molar occupancy, labile copper, species-resolved release, and mass-balanced permeation. The chemical/formulation axis is therefore partly an appraisal of reporting quality rather than underlying experimental quality, and some studies classified as C0–C1 may have used well-characterized material that was not adequately described. The B/C framework itself is author-defined, has not been externally validated, and is not intended to replace a formal risk-of-bias instrument for a future indication-specific systematic review. Likewise, the stage-gated roadmap and provisional decision specifications are hypotheses for disciplined development, not consensus standards. These limitations reinforce the case for the standardized reporting set proposed in Section 3.

## 12. Conclusions

GHK-Cu is neither a proven therapeutic nor merely a marketing construct. It is a chemically dynamic Cu(II)-peptide system with a reproducible preclinical profile in matrix remodeling, epithelial repair, inflammation/redox biology, and angiogenesis, but the evidentiary meaning of “GHK-Cu” depends on which entity was actually studied. Apo-GHK, canonical GHK-Cu, GHK-derived copper peptides, and unrelated copper-peptide systems cannot be treated as interchangeable. The human record remains small: historical cosmetic studies are chemically undercharacterized, the 13-participant post-CO2-laser study was negative on objective endpoints [26], the 18-participant 2026 eyebrow study provides a positive controlled cosmetic signal but remains C1 for active-entity characterization [47], and the ongoing phase 2 wound trial had no efficacy results at the review cutoff [48]. Accordingly, the registration of NCT07437586 indicates ongoing clinical investigation but cannot be interpreted as evidence of clinical efficacy or successful clinical translation.

The central pharmaceutical argument is therefore definitional. By “active pharmaceutical entity” we mean the reproducible chemical state intentionally administered and linked to pharmacology, not an immutable molecule or an established regulatory API designation. For canonical GHK-Cu, a credible control strategy requires peptide identity, total peptide and copper, molar occupancy, a bounded labile copper fraction, orthogonal speciation, related-species control, and mechanism-linked potency. If a formulation instead works through controlled copper transfer or an in situ transformation product, the active entity should be redefined accordingly. Separate assay of total peptide and total copper is necessary but cannot answer that mechanistic question.

The move from creams toward liposomes, microneedles, responsive polymers, self-assembling/photo-crosslinked hydrogels, electrospun dressings, and injectable depots is a genuine opportunity to treat GHK-Cu as a drug-delivery cargo, but it also amplifies attribution risk. Several high-performing systems use GHK-derived dimers or other copper-peptide constructs rather than canonical monomeric GHK-Cu, and multifunctional carriers can reproduce part of the apparent biological effect. Factorial designs with blank-carrier, apo-peptide, equimolar free copper, and free complex arms at matched exposure are therefore needed before carrier performance is attributed to GHK-Cu itself.

What the field needs is not additional demonstrations that a nominal copper-peptide formulation changes a biomarker but a reproducible chain from defined entity to species-resolved exposure, causal pathway engagement, functional tissue effect, and then controlled human benefit. The stage-gated sequence proposed here is explicitly an author-derived framework for organizing that chain, not a generalized development standard. Its value will ultimately depend on whether future studies report enough coordination chemistry and formulation data to test, refine, or reject its individual gates.

## Figures and Tables

**Table 1:** Systematic study-level comparison of major GHK-Cu and copper-peptide delivery systems, with entity class (E1–E4) shown explicitly.

Study/ Formulation	Claimed Loading	Molar Occupancy?	Labile Cu?	Species- Resolved Release?	Factorial Controls?	Primary Objective Outcome	Stability/ Sterility/GMP
E2|Dymek 2023 [27] GHK-Cu liposomes	0.5 mg/mL feed; EE 20.0–31.7%	NR; nominal complex	NR	No; bulk/dialysis release	Partial; no complete apo-GHK/free Cu/carrier set	Size, EE, release; tyrosinase/elastase assays	Short-term colloidal stability; no ICH-complete stability, validated sterility, or GMP scale-up
E2|Li 2015 [28] solid MN pretreatment + GHK-Cu	No carrier loading; solution applied after MN	NR	NR	No; peptide and Cu measured separately	Partial; intact skin vs. MN-treated	Ex vivo human/porcine permeation; irritation	Experimental device testing; no product-level ICH/GMP validation
E2|Sharma 2019 [29] pH-sensitive GHK-Cu superabsorbent polymer	Formulation mass loading; not intact-complex molarity	NR	NR	No; bulk controlled release	Partial; no full four-arm attribution	Controlled release; in vitro/in vivo wound healing	No ICH-complete stability, validated sterilization cycle, or GMP package
E2|Sharma 2022 [30] responsive GHK-Cu polymer gel	Formulation content reported	NR	NR	No; bulk release kinetics	Partial; carrier effects not fully separated	Controlled release; wound-healing performance	No ICH-complete stability or validated sterilization/GMP process
E3|Lee 2023 [31] photo-crosslinked HA/GHK nanofiber-Cu hydrogel	GHK nanofiber content + Cu complexation	Analytical complexation; no quantified occupancy	NR	No; intact vs. exchanged Cu not resolved	Partial; no full apo-GHK/free Cu set	Fibroblast response, collagen, angiogenesis, wound closure	Photo-crosslinking characterized; no registration stability, validated sterility, or GMP scale-up
E3|Cong 2025 [32] ROS-responsive dimeric Cu-peptide hydrogel	Dimeric peptide; nominal GHK:Cu^2+^ 1:0.5	Nominal + UV-Vis/EPR; occupancy NR	NR	Partial; D-CuP release, but apo/labile Cu not concurrent	Strong but incomplete; no equimolar free Cu arm	Protease stability; cellular effects; infected diabetic-wound closure	Preclinical stability/biocompatibility; no ICH-complete shelf-life, validated sterility, or GMP validation
E2 + co-active|Wang 2024 [34] electrospun PVB/PVP + GHK-Cu/Pionin	Dual-drug loading; occupancy not quantified	NR	NR	No; drug/bulk release only	Incomplete; Pionin + polymer confound attribution	Antioxidant, anti-inflammatory, antimicrobial, wound-healing outcomes	Fiber/release characterized; no validated sterility, ICH-complete stability, or GMP scale-up
E2|Hu 2025 [35] GHK-Cu/HAP microsphere injectable	Electrostatic HAP loading; ~7-day release	NR	NR	No; nominal/total GHK-Cu release	Partial; no apo-GHK + equimolar free Cu set	Injectability, release, inflammatory/redox markers, collagen	Injectability shown; no validated sterility, ICH-complete stability, depot-speciation comparability, or GMP validation

Abbreviations: Cu, copper; EE, encapsulation efficiency; GMP, good manufacturing practice; HA, hyaluronic acid; HAP, hydroxyapatite; ICH, International Council for Harmonisation; MN, microneedle; PVB/PVP, poly(vinyl butyral)/poly(vinyl pyrrolidone). “NR” denotes that the requested active-entity measurement was not reported.

**Table 2:** Dual-axis evidence map for major GHK-Cu-associated pharmacological claims. Biological evidence level (B1–B5) and chemical/formulation quality (C0–C3) are graded independently.

Claim	Representative Evidence/Entity Class	Biological Evidence (B)	Chemical/Formulation Quality (C)	Key Limitation/Decisive Next Experiment
Matrix remodeling	Canonical/nominal GHK-Cu (E2): fibroblast and wound models show collagen, GAG, MMP, and proteoglycan modulation [3,4,5,6,7].	B3–B4	C1	Standardize species and measured exposure; demonstrate improved matrix architecture and mechanics, not only biomarker increases.
Re-epithelialization	Canonical/nominal GHK-Cu (E2): keratinocyte integrin and p63 changes [9].	B4	C1	Confirm barrier restoration and migration in human skin models with apo-GHK, free copper, and vehicle controls.
Anti-inflammatory activity	Canonical GHK-Cu (E2) in mouse lung injury/silicosis and zebrafish models [11,39,40].	B3–B4	C1–C2	Establish route-specific exposure-response and replicate with pharmaceutical-grade active-entity characterization.
Antioxidant/carbonyl control	Primarily apo-GHK chemistry (E1) plus mixed GHK/GHK-Cu cellular evidence [18,19,20].	B4	C0–C1 for GHK-Cu attribution	Demonstrate that intact, characterized GHK-Cu produces the same protection at clinically achievable exposure and separate peptide from copper effects.
	GHK-derived or mixed copper-peptide biomaterials (E3/E4) rather than clean canonical GHK-Cu comparisons [32,41].	B3–B4	C0–C1 for canonical GHK-Cu attribution	Quantify mature perfused vessels and use entity-matched controls before transferring the effect to canonical GHK-Cu.
Cosmetic skin remodeling	Human topical reports (E2 nominal) include small, conference-level, or multicomponent studies [23,24,25].	B1–B2/B5	C1	Independent randomized dose-ranging study with defined active entity, vehicle control, objective imaging, and local exposure.
Postprocedure repair	Controlled topical study with nominal copper tripeptide (E2) did not show clear objective benefit [26].	B1	C1	Replicate with adequate power, defined occupancy/speciation, objective endpoints, and mechanism-matched timing.
Systemic regenerative claims	Mixed E1/E2 evidence: apo-GHK transcriptomic work, GHK-Cu animal studies, and lower-organism models [10,11,12,13,39,40,41,42,43].	B3–B5	C0–C2 depending on study	Require route-appropriate PK/biodistribution, active-entity confirmation, toxicology, and exposure-linked efficacy before clinical extrapolation.

Abbreviations: GAG, glycosaminoglycan; MMP, matrix metalloproteinase; PK, pharmacokinetics.

**Table 3:** Pharmaceutical identity and developability profile of GHK-Cu.

Attribute	Pharmaceutical Relevance	Minimum Characterization Expected
Chemical identity	The nominal 1:1 Cu(II):GHK complex is not sufficient to describe all species present in solution.	Peptide sequence and purity; counterion; Cu:peptide molar ratio; water content; mass balance.
Coordination/speciation	pH, ionic strength, competing ligands, and concentration can alter charge and the distribution of monomeric or higher-order species.	Orthogonal spectroscopic or MS evidence across formulation pH; free Cu^2+^ assay; speciation stress study.
Potency	Total peptide concentration may not predict biological activity when copper occupancy varies.	Validated cell-based or biochemical assay linked to the intended mechanism, alongside chemical content.
Proteolytic stability	The tripeptide may be rapidly degraded in biological fluids; stability depends on route and matrix.	Intact-complex and peptide-degradation profile in skin, wound fluid, plasma, or relevant simulated media.
Permeation/retention	Hydrophilicity limits passive flux, whereas ionization and complexation can alter partitioning.	Mass-balanced ex vivo deposition and receptor-phase recovery; intact-species confirmation.
Oxidation/reactivity	Copper can participate in redox chemistry; excipients and antioxidants may be protective or may change speciation.	Oxidative stress panel, peroxide sensitivity, container-closure compatibility, free copper trend.
Aggregation/particles	Peptide-related particles are especially important for injectable products.	Subvisible particles, size-exclusion or equivalent aggregate method, stress-induced aggregation.
Microbiology/sterility	Wound and injectable products require route-appropriate microbial control.	Bioburden, preservative effectiveness or sterility, endotoxin, and packaging integrity.

**Table 4:** Comparison of GHK-Cu with representative copper-binding and regenerative peptides.

Peptide/Complex	Copper Coordination and Origin	Principal Regenerative Rationale	Feature Distinguishing It from GHK-Cu
GHK-Cu (reference)	Endogenous tripeptide; 1:1 Cu(II) via histidine imidazole, deprotonated backbone amides, and amino terminus	Sequence-specific dual action on matrix synthesis and turnover, with copper-dependent antioxidant and angiogenic effects	Reference entity: best-characterized coordination and broadest multimodal dataset; principal liability is labile copper control
AHK-Cu (alanyl-histidyl-lysine–Cu)	Synthetic; histidine-anchored Cu(II) coordination	Cosmetic angiogenesis, hair and skin claims	Shares the motif but rests on a thin, poorly characterized evidence base
Dimeric copper peptide [32]	Engineered GHK-derived dimer; multivalent Cu(II)	Protease-stable diabetic-wound repair from a responsive hydrogel	A derivative with distinct pharmacokinetics, stability, and safety; not interchangeable with monomeric GHK-Cu
Food-derived tripeptide–Cu [33]	Dietary-sequence tripeptide; coordinated Cu(II)	Self-healing hydrogel for infected wounds	Shows copper coordination is a class property, not GHK-specific
ATCUN/DAHK-type (albumin N-terminus)	Endogenous; very high-affinity square-planar Cu(II)	Copper buffering and redox silencing rather than proregenerative signaling	Tight chelation suppresses the labile copper GHK-Cu is proposed to exploit
Metal-free matrikine peptides (e.g., palmitoyl pentapeptide, GEKG)	No metal cofactor	Sequence-directed extracellular-matrix synthesis	Avoid copper redox liabilities but forgo copper-dependent enzymatic and angiogenic contributions

Abbreviations: AHK, alanyl-histidyl-lysine; ATCUN, amino-terminal Cu(II)- and Ni(II)-binding motif; DAHK, aspartyl-alanyl-histidyl-lysine; GEKG, glycyl-glutamyl-lysyl-glycine.

**Table 5:** (**A**) Human clinical evidence for topical/local GHK-Cu or copper-tripeptide products, separated from preclinical evidence. (**B**) Therapeutic domains ordered by biological evidence level. Chemical/formulation quality is interpreted independently using the C0–C3 framework in Table 2.

(**A**)					
**Human Study/** **Status**	**Design and** **Sample**	**Test Material**	**Primary** **Endpoint(s)**	**Result**	**Pharmaceutical** **Interpretability**
Abdulghani et al. 1998 [24]	Comparative topical human study; small cohort	Copper-binding peptide cream in a multicomponent comparison	Skin ultrastructure/remodeling markers	Reported ultrastructural differences; not a modern efficacy trial	C1: nominal copper-peptide product; occupancy, labile Cu, speciation, and local exposure not reported
Leyden et al. 2002 [25]	Conference-level cosmetic report	Copper-peptide facial cream	Cosmetic skin outcomes	Positive cosmetic signal reported	C1/limited reporting; conference-level evidence and active entity not chemically resolved
Miller et al. 2006 [26]	Randomized post-CO2-laser comparison; 13 completers	Topical copper-tripeptide regimen	Erythema, wrinkles, skin quality; blinded/objective assessments	No significant objective advantage; satisfaction favored copper-tripeptide arm	C1: active content/speciation not characterized; underpowered for moderate effects
Bo et al. 2026 [47]	Randomized, double-blind, vehicle-controlled split-face study; 18 participants	Nominal 2% GHK-Cu serum	Eyebrow hair count, diameter, photographic assessment, satisfaction	Improvement on treated side at 12 weeks; no serious adverse events reported	C1: concentration reported, but occupancy, labile Cu, speciation, and tissue exposure not demonstrated
NCT07437586 [48]	Phase 2 randomized, double-blind, vehicle-controlled split-wound study; ongoing	Topical GHK-Cu gel	Acute-wound re-epithelialization	No efficacy results available at cutoff	Registry record only; cannot support efficacy or clinical translation until results are reported
(**B**)					
**Domain**	**Highest Biological** **Evidence Level (B)**	**Principal Confounder**	**Decisive Next Study**		**Realistic Near-Term Positioning**
Photoaging/cosmetic remodeling	B2–B5: small pilot, conference and multicomponent product studies [13,23,24,25]	Undefined active content; multicomponent vehicles; no exposure data	Randomized, vehicle-controlled dose ranging with objective imaging and confirmed cutaneous exposure		Cosmetic claim substantiation; medicinal claims not currently supportable
Postprocedural repair/acute wounds	B1: one small controlled study negative on objective endpoints [26]; a phase 2 vehicle-controlled trial is recruiting [48]	Underpowered trial; uncharacterized product; possible endpoint–mechanism mismatch	Adequately powered trial after standardized ablative procedure with defined complex content and blinded objective primary endpoint		Highest-value near-term clinical target; standardized injury and short follow-up
Chronic and diabetic wounds	B3–B4, carrier-dependent [29,30,32,33,34]	All effective systems are multifunctional; carrier and coactive effects not separated	Factorial in vivo dose ranging with blank carrier, apo-peptide, free copper and free complex arms, then exploratory human study with wound-fluid speciation		Strong scientific rationale; requires attribution work before clinical investment
Implant, scaffold and hard tissue	B4 [41,46,49]	Unclear whether intact complex or slow copper release is the operative mechanism	Interface-level species-resolved release and target-engagement measurement		Attractive device or combination-product route; low systemic exposure
Injectable aesthetic/soft-tissue depot	B3–B4 [35]	Filler volume and mechanical effects mimic biological improvement; irreversibility	Preclinical study with sham filler, blank microsphere, free complex and free copper arms plus full injectable quality package		Commercially attractive but highest evidentiary and quality burden
Pulmonary, neurological and systemic	B3–B5: mixed apo-GHK computational evidence and GHK-Cu rodent/lower-organism studies [10,11,12,13,39,42,43]	Apo-peptide and computational signatures conflated with formulated complex; no human exposure data	Route-appropriate pharmacokinetics, biodistribution and repeat-dose toxicology		Exploratory research only; must not be used to support dermal claims

**Table 6:** Route-specific analytical and safety expectations for GHK-Cu products.

Attribute or Study	Topical Cosmetic	Topical or Dermal Medicinal Product	Wound Dressing (Device or Combination)	Injectable or Implanted Product
Intact-complex assay	Recommended	Required, stability-indicating	Required, stability-indicating	Required, stability-indicating, and orthogonal
Labile copper limit	Recommended; <=5 mol% total Cu as provisional development specification	Required; <=5 mol% total Cu, tox-justified	Required; <=5 mol% total Cu in product and biorelevant release	Required; <=1 mol% total Cu at release and during depot release; tox-driven tightening
Speciation across formulation pH	Recommended	Required	Required	Required
Potency assay linked to mechanism	Optional	Required	Required where medicinal action claimed	Required
Species-resolved release testing	Not applicable	Required for modified-release systems	Required in biorelevant medium	Required, plus local and systemic copper
Mass-balanced ex vivo permeation	Recommended	Required	Situational	Not applicable
Excipient compatibility with redox and binding endpoints	Recommended	Required	Required	Required
Microbiological control	Preservative efficacy	Preservative efficacy or sterility	Bioburden or sterility, packaging integrity	Sterility, endotoxin, particulates
Irritation, sensitization, phototoxicity	Required	Required	Required	Situational
Local histopathology and depot behavior	Not applicable	Situational	Situational	Required
Angiogenic safety assessment	Not applicable	Situational	Recommended	Required
Molar occupancy for nominal 1:1 GHK-Cu	Target 0.95–1.05	Target 0.95–1.05; required for medicinal claim	Target 0.95–1.05 before loading and after release	Target 0.95–1.05 with tight batch/site comparability
Systemic copper exposure screen	Worst-case estimate for large-area/repeated use	Proposed early trigger: product-derived absorbed Cu < 0.5 mg/day unless TK justifies more	Proposed early trigger: absorbed Cu < 0.5 mg/day for chronic wounds unless TK justifies more	Do not derive from oral limits; route-specific repeat-dose TK/NOAEL and accumulation studies required

**Table 7:** Author-proposed stage-gated decision framework for a GHK-Cu local-delivery program; not a consensus or regulatory development standard.

Gate	Question	Minimum Deliverable	Stop or Redesign Criterion
0. Entity	What exactly is the active?	Characterized complex; molar occupancy; validated labile copper method and provisional limit; potency assay; standardized reporting set	Labile copper not controllable across intended formulation pH range
1. Formulation	Can a product hold and release it intact?	Prototype with defined loading/distribution; species-resolved biorelevant release; 3-month accelerated stop/go checkpoint with full active-entity panel; excipient compatibility. Continue to ICH-complete stability for medicinal development	Predominant labile copper release, occupancy/speciation drift, loss of complex integrity, or non-scalable/sterilization-sensitive process
2. Attribution	Is the effect the peptide, the copper, or the carrier?	Factorial dataset versus blank carrier, apo-peptide, equimolar free copper and free complex at matched activity; exposure–response for one mechanism-linked endpoint	Blank carrier reproduces most of the effect; redirect as a biomaterials program
3. In vivo	Does measured local exposure produce a functional effect?	Indication-matched model; local intact-complex concentration over the dosing interval; functional endpoint; angiogenic and oxidative safety screens	Efficacy only at exposures a human formulation could not achieve
4. Human exploratory	Does it engage the pathway in people, safely?	Small vehicle-controlled study; biopsy or imaging biomarker with prespecified pathway module; local exposure confirmation; tolerability	No exposure-linked pathway engagement, or unacceptable local tolerability
5. Confirmatory	Does it change a clinically meaningful outcome?	Adequately powered indication-specific trial with objective primary endpoint and mechanism-matched endpoint timing	Not applicable; this gate is the decision point for registration

## Data Availability

No new data were generated or analyzed in this study. All information is derived from previously published studies, which are cited within the manuscript.

## Abbreviations

The following abbreviations are used in this manuscript:

CPP	Critical process parameter
CQA	Critical quality attribute
EE	Encapsulation efficiency
GAG	Glycosaminoglycan
GHK	Glycyl-L-histidyl-L-lysine
GHK-Cu	Copper(II)-coordinated glycyl-L-histidyl-L-lysine
MMP	Matrix metalloproteinase
PDI	Polydispersity index
PK	Pharmacokinetics
ROS	Reactive oxygen species
TIMP	Tissue inhibitor of metalloproteinases
EFSA	European Food Safety Authority
GMP	Good manufacturing practice
ICH	International Council for Harmonisation
NOAEL	No-observed-adverse-effect level
TK	Toxicokinetics
